# Antihypertensive drug targets and breast cancer risk: a two-sample Mendelian randomization study

**DOI:** 10.1007/s10654-024-01103-x

**Published:** 2024-02-24

**Authors:** Guoqiao Zheng, Subhayan Chattopadhyay, Jan Sundquist, Kristina Sundquist, Jianguang Ji

**Affiliations:** 1 Center for Primary Health Care Research, Lund University/Region Skåne, Jan Waldenströms Gata 35, 205 02 Malmö, Sweden; 2 Division of Clinical Genetics, Department of Laboratory Medicine, Lund University, Lund, Sweden; 3 Department of Family Medicine and Community Health, Department of Population Health Science and Policy, Icahn School of Medicine at Mount Sinai, New York, NY USA; 4 Center for Community-Based Healthcare Research and Education (CoHRE), Department of Functional Pathology, School of Medicine, Shimane University, Matsue, Japan

**Keywords:** Antihypertensive medication, Cancer risk, Causal association, Drug repurposing, Adverse drug effect

## Abstract

Findings on the correlation between the use of antihypertensive medication and the risk of breast cancer (BC) have been inconsistent. We performed a two-sample Mendelian randomization (MR) using instrumental variables to proxy changes in gene expressions of antihypertensive medication targets to interrogate this. Genetic instruments for expression of antihypertensive drug target genes were identified with expression quantitative trait loci in blood, which should be associated with systolic blood pressure to proxy for the effect of antihypertensive drug. The association between genetic variants and BC risk were obtained from genome-wide association study summary statistics. The summary-based MR was employed to estimate the drug effects on BC risk. We further performed sensitivity analyses to confirm the discovered MR associations such as assessment of horizontal pleiotropy, colocalization, and multiple tissue enrichment analyses. The overall BC risk was only associated with *SLC12A2* gene expression at a Bonferroni-corrected threshold. One standard deviation (SD) decrease of *SLC12A2* gene expression in blood was associated with a decrease of 1.12 (95%CI, 0.80–1.58) mmHg of systolic blood pressure, but a 16% increased BC risk (odds ratio, 1.16, 95% confidential interval, 1.06–1.28). This signal was further observed for estrogen receptor positive (ER +) BC (1.17, 1.06–1.28). In addition, one SD decrease in expression of *PDE1B* in blood was associated with 7% decreased risk of ER + BC (0.93, 0.90–0.97). We detected no evidence of horizontal pleiotropy for these associations and the probability of the causal variants being shared between the gene expression and BC risk was 81.5, 40.5 and 66.8%, respectively. No significant association was observed between other target gene expressions and BC risk. Changes in expression of *SLC12A2* and *PDE1B* mediated possibly via antihypertensive drugs may result in increased and decreased BC risk, respectively.

**Supplementary Information:**

The online version contains supplementary material available at 10.1007/s10654-024-01103-x.

## Background

Nearly one in three adults aged 30–79 years were estimated to have hypertension globally in 2019 [1], which led to a high prevalence of antihypertensive medication use. It is the most commonly prescribed medication in Sweden with over 2.1 million patients taking anti-hypertensive drugs in 2020 [2]. The exposure to antihypertensive medication has also been increasing in recent years. In UK, the prevalence of primary care patients with antihypertensive drug prescriptions has increased from 7.8% (1988) to 21.9% (2018) [3]. These drugs are usually prescribed to hypertensive patients as a long-term management [4]. Given the large disease burden and high consumption of the antihypertensive medication, there are concerns surrounding their carcinogenic potential as well as interests regarding their possible anti-cancer effect since the targets of these drugs are widely distributed in normal tissue and may be involved in tumor development. For example, renin-angiotensin system, the target for angiotensin-converting enzyme inhibitors (ACEi) and angiotensin receptor blockers (ARBs), contains components that promote or inhibit cellular proliferation [5]. Many observational studies with cohort or case–control design have evaluated the possible link between antihypertensive medication and risk of breast cancer (BC) [6–8], the most common type of cancer and the leading cause of cancer death among women worldwide [9]. However, the results are conflicting, as these studies suffer from various biases, including unmeasured confounding, immortal time bias, confounding by indication and surveillance bias, which might result in inconsistent conclusions. Due to such shortcomings of the observational studies, previous results could not draw causative conclusions. In addition, there are ethical constraints for conducting randomized clinical trials (RCTs). Therefore, we opted for an alternative design to investigate the causal effect of anti-hypertensive medications on BC risk.

Mendelian randomization (MR) mimics a natural experiment by using genetic variants as a proxy (instrumental variable) for the modifiable exposure [10]. The principle is that randomization occurs naturally at conception when genetic variants are allocated at random to individuals from their parents. Consequently, the inherited genetic variants are independent of potential confounding environmental exposures. MR can be regarded as analogous to an RCT that uses genetic variation as the method of randomization ultimately providing causal insights. In addition, the risk estimated from MR reflects a lifetime risk, which is longer than the follow-up in a RCT. MR has successfully identified unintended drug effects including adverse drug effects and drug repurposing [11, 12]. As for the association between antihypertensive medication and cancer risk, Yarmolinsky et al. reported long-term ACE inhibition to be associated with an increased risk of colorectal cancer by using a MR analysis [13]. However, the ARB, a common antihypertensive medication [14], was not included in the analysis. Additionally, the instrumental variable for each class of drug was selected based on only one target. However, antihypertensive drugs may act on multiple targets involved in different pathways, thus analyses capturing all the possible targets with corresponding instrumental variables for each drug are needed. Finally, the instruments for the target from Yarmolinsky et al. were derived only from the blood serum, whereas the targeted genes may be differentially expressed in a tissue-specific manner [15].

Here we aimed to investigate the effect of antihypertensive medication use on BC risk using a two-sample MR design with a consideration of all the commonly prescribed medications for hypertension, and exploring the eQTL from both the whole blood and several other tissues. This study will add evidence to the current knowledge derived from observational studies and try to draw causal conclusions regarding the potential association between antihypertensive medication use on BC risk.

## Methods

**Fig. 1:**
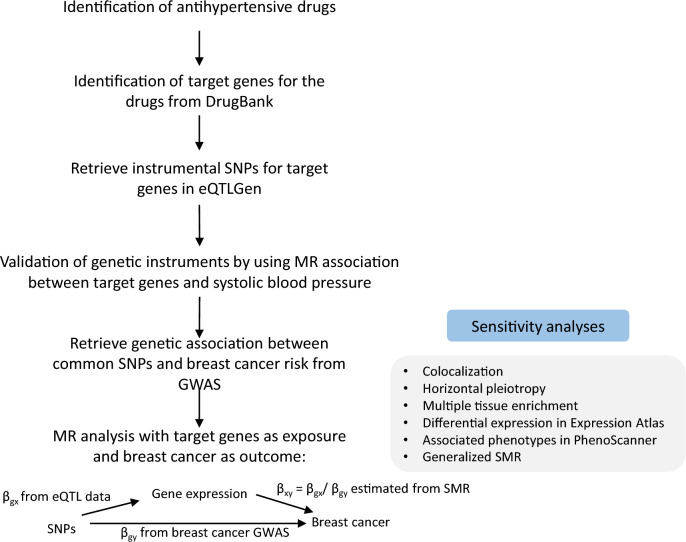
The flowchart of the study. *x* represents gene expression (exposure). *y* represents breast cancer risk (outcome). *g* represents genetic variants (instrumental variable). β_gx_ is the effect size of the association between genetic variants and target gene expression (the exposure). β_gy_ is the effect size of the association between genetic variants and breast cancer risk (outcome). β_xy_ is the effect size of the association between target gene expression and breast cancer risk, estimated with β_gx_/ β_gy_ using SMR. *SNP* single-nucleotide polymorphism, *MR* Mendelian randomization, *GWAS* genome wide association study, *SMR* summary-based MR, *eQTL* expression quantitative trait loci

This study was based on several publicly available databases, which are summarized in the Supplementary File 1a, where we used the summary statistics without accessing any personal information. The study design is demonstrated in Fig. 1, which is also described below. We used two-sample MR to estimate the association between target gene expression (the exposure, say x) and BC risk (the outcome, say y) with effect size β_xy_. The basic principle of MR is that, if a modifiable exposure alters outcome then the instrumental variable (here genetic variants, say g) that modifies the level of that exposure, should also be related to the outcome [16]. Expression quantitative trait loci (eQTL) data were used to assess the association between genetic instruments and level of target gene expressions (β_gx_). The associations between genetic instruments and BC risk were estimated with genome wide association study (GWAS) summary statistics (β_gy_). In a MR analysis, β_xy_ (defined as β_xy_ = β_gy_/β_gx_) is interpreted as the effect of x on y free of non-genetic confounders. For a genetic instrument to be considered valid three basic assumptions must have held: (1) The genetic variants are associated with the exposure (relevance assumption); (2) There are no unmeasured confounders of the associations between genetic variants and outcome (independence assumption); (3) The genetic variants affect the outcome only through their effect on the exposure (exclusion restriction) [10]. We further performed sensitivity analyses to confirm the discovered MR associations such as assessment of pleiotropy, colocalization, and multiple tissue enrichment analyses.

### Identification of drug target genes

The commonly prescribed antihypertensive medications, including ACEis, ARBs, beta-blockers (BB), calcium channel blockers (CCB), diuretics and other antihypertensive agents, were included for analysis [14]. We identified the genes targeted by these different classes of anti-hypertensive medication through DrugBank [17].

### Discovery of genetic instruments for target gene expression

To identify common single-nucleotide polymorphism (SNPs, population minor allele frequency > 1%) associated with the target gene expression of antihypertensive drug in whole blood, we extracted publicly available eQTL data (both genders) from eQTLGen (https://www.eqtlgen.org/). The consortium incorporates 37 datasets, with a total of 31,684 individuals of both genders and reports on 16,987 genes expressed in whole blood. The whole blood was used as the tissue for the main analysis because it is an easily accessed tissue. The eQTL SNPs for this analysis were from *cis*-regulated regions (1 MB on either side of a gene) with default *p*-value 5.0e–8. We used F statistic to assess the strength of the instrumental SNPs.

### Validation of genetic instrument with systolic blood pressure GWAS

In order to have valid instruments for the medication of interest (blood pressure-lowering medication), we performed a two-sample MR analysis with target gene expression in blood (using the eQTL data) as exposure and systolic blood pressure (SBP) as the outcome. SBP was chosen as it is relatively more important in the management of hypertension compared to diastolic blood pressure [18]. The summary statistics for SBP was a GWAS of SBP in 757 601 individuals of European ancestry (male and female) drawn from UK Biobank and the International Consortium of Blood Pressure Genome Wide Association Studies (ICBP) [19]. The summary-based MR (SMR) method (version 1.02) was used to perform the two-sample MR analysis [20]. For SNPs that passed the significance threshold for the SMR test (i.e., *p* < 0.05). Additionally, we performed the HEIDI (heterogeneity in dependent instruments) test to distinguish pleiotropy from linkage with p-value threshold of 0.01, in which up to top 20 SNPs by default were used for heterogeneity test with linkage disequilibrium (LD) pruning (0.05–0.9) [20].

### Accumulation of genetic summary statistics for breast cancer risk

We obtained publicly available GWAS summary statistics for BC risk among women from the Breast Cancer Association Consortium (BCAC) [21]. The analysis for the overall BC risk included 118,474 cases and 96,201 controls of European ancestry participating in 82 studies. For analyses of BC molecular subtypes, 7325 participants were included for luminal A-like cases, 1779 for luminal B/HER2-negative-like cases, 1,682 for luminal B-like cases, 718 for HER2-enriched-like, 2006 for triple-negative cases and 20,815 for controls [21]. We also obtained the GWAS summary statistics for estrogen receptor-positive (ER+) and −negative (ER−) BC from BCAC, which contained 69,501 ER+BC cases, 21,468 ER-BC cases and 105,974 controls [22].

### MR analysis between target gene expression in blood and breast cancer risk

We performed SMR analysis to estimate the association between target gene expression change (using whole blood eQTL) and BC risk (using GWAS). As the SMR results were based on the association of the top SNP, we additionally performed multiple SNPs-based SMR (SMR-multi) by including numerous associated SNPs at an eQTL locus in the SMR analysis, which may increase the power of the test [23]. The default value, 0.1 for LD *r*^2^ threshold was used to prune SNPs (eQTLs) in the SMR-multi test. The HEIDI test was also performed with SMR by default. Bonferroni correction was used to identify significant associations due to multiple testing. For the association that reached corrected significant level, we generated SMR locus plot using the method presented on SMR webpage (https://yanglab.westlake.edu.cn/software/smr/#Overview).

### Sensitivity analyses

#### Colocalization analysis

This analysis was to assess if two independent association signals at the same locus, typically generated by two GWAS studies, are consistent with a shared causal variant. In the context of our study, we examined if the drug target gene expression and occurrence of BC shared a common causal variant in a given region by applying a Bayesian localization approach [24]. As a convention, a posterior probability larger than 0.80 was considered supportive for a common causal variant. The R package ‘coloc’ (v3.1, https://cran.r-project.org/web/packages/coloc/) was used to perform the test [24]. Given ‘coloc’ assumes a single causal variant, we also performed ‘coloc.SuSiE’ considering the existence of multiple causal variants. However, no credible sets were found for BC possibly due to the relatively high *p* value in BC GWAS [25]. Thus, we only presented the results based on single causal variant according to the recommendation provided by the author of ‘coloc.SuSiE’ [25].

#### Assessment of horizontal pleiotropy

Horizontal pleiotropy occurs when any effects of the genetic variant on the outcome are through pathways other than via the exposure of interest (here target gene expression). It can distort MR tests, leading to inaccurate causal estimates, loss of statistical power and potential false-positive causal relationships. We tested horizontal pleiotropy by extracting available associations with all other nearby genes (within a 2 MB window) for each genetic instrument. Only cis-genes were considered for the analyses due to the proximity to the gene of interest and biological relevance conferred owing to gene expression. For the nearby genes showing significant association with the genetic instrument, we performed SMR analysis to test if the expression of these genes was associated with BC risk. For those genes with significant SMR associations, colocalization analysis was performed to estimate the probability that two association signals shared the same causal variant.

#### Multiple tissue sensitivity

We then evaluated the impact of the actionable targets in several different tissues along with whole blood and breast epithelium. Gene set enrichment analysis was performed with the assorted drug target genes to quantify all biological pathways prone to be affected by the drugs. RNA seq data on different tissues (except prostate and testis) were utilized for this purpose and analyzed with the R package ‘TissueEnrich’ [26]. The enrichment analysis queried expression modulation with fold changes across different human tissue types. A significant departure was defined at an adjusted fold change in expression of value 2 and the fold change was also tested for statistical significance. For tissues that showed significant fold changes, further MR association between tissue-specific gene expression and BC risk were assessed using GTEx (V8) cis-eQTL summary statistics (https://yanglab.westlake.edu.cn/data/SMR/GTEx_V8_cis_eqtl_summary.html). The cut-off used to identify eQTLs in GTEx was the same as the one for blood. We listed breast epithelium separate from other tissue because it is the most relevant for breast cancer.

#### Differential gene expression in expression atlas

To further analyze potential biological implications of the causal variants, we retrieved transcriptomic data of the significant genes for tumor tissue (or other tissue from BC patients) and normal tissue (or other tissue from healthy controls) on Expression Atlas (https://www.ebi.ac.uk/gxa/home). Differential gene expressions between tissues were evaluated as fold change using normal tissue (or other tissue from healthy controls) as reference.

#### Phenotypes associated with the top SNPs of significant genes in PhenoScanner

We identified the traits and diseases that were associated with the causal variants in PhenoScanner (v2) for the significant genes and checked the relevance of these phenotypes with breast cancer risk from literature. The traits were retrieved based on *p* value less than 5.0e–8, and *r*^2^ > 0.8 with European ancestry.

#### MR analysis to estimate the association of SBP with breast cancer

To determine whether the association between drug target gene expression and BC risk was likely to be mediated via changes in blood pressure or whether the association may be driven independently, we estimated the effect size for the association between genetically estimated SBP and BC using the generalized summary data-based MR (GSMR) method [27]. The GSMR (implemented in Genome-wide Complex Trait Analysis, version 1.91.7) is an extension of SMR that uses multiple genetic variants associated with the exposure to test for potential causality. The above-mentioned GWAS summary statistics on SBP (exposure) and overall BC risk (outcome) in European individuals were used for the analysis. As usual, a HEIDI *p* < 0.01 was used to detect outlying variants. In addition, we performed two-sample MR analysis using the R package Two-Sample MR to check the consistency of the signals from the two different methods. Two-sample MR were performed with consideration of LD of the instrumental SNPs.

## Results

### Genetic instrument selection and validation

**Fig. 2:**
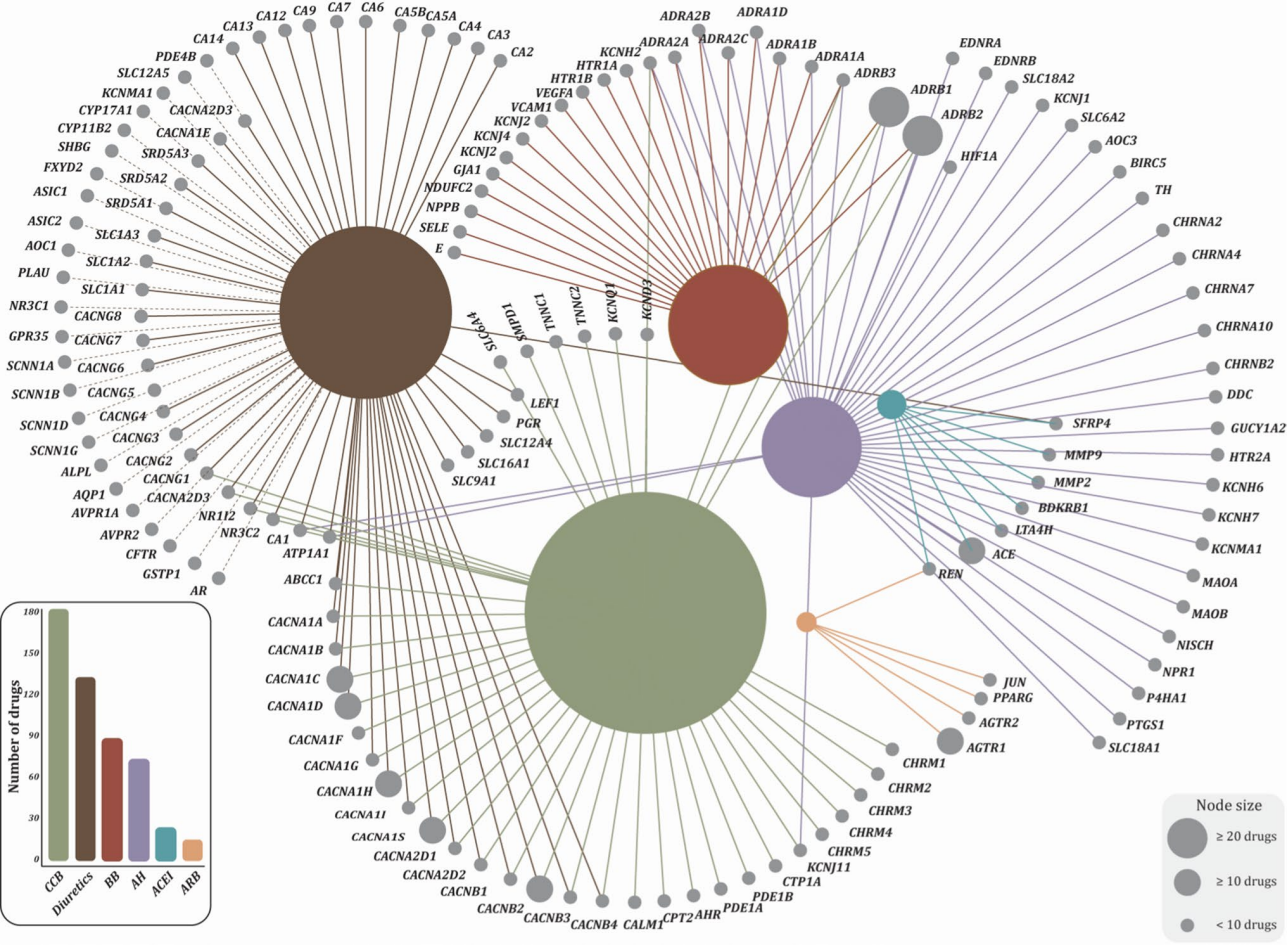
A network of established links between hypertension drugs considered and the corresponding target genes. Each color represents a different class of drugs (annotated in the histogram) and the circle size represents the number of drugs belonging to a class. *CCB* calcium channel blockers, *BB* beta-blockers, *AH* antihypertensives, *ACEi* angiotensin-converting enzyme inhibitors, *ARBs* angiotensin receptor blockers

We identified a total of 164 blood pressure modulatory drugs from the WHO Collaborating Centre for Drug Statistics Methodology (https://www.whocc.no/). In DrugBank database, 124 of them were identified to target a total of 154 genes. There were expectedly several overlaps between drug classes and associated targets (Fig. 2, details in Supplementary File 1b). For example, *SLC12A2* can be targeted by quinethazone, bumetanide and torasemide, and at the same time, torasemide can also target on gene *SLC12A1*. Among the 154 chosen targeted genes, 72 genes were identified to have strong association with the eQTL SNPs in eQTLGen (*F* statistic > 10, P_eQTL_ < 5e–8), among which, 32 were found to be causally associated with SBP with statistical significance (*p* < 0.05). A total of 23 associations were confirmed by the HEIDI outlier test (p_HEIDI > 0.01), which were further considered in the MR analysis with BC as the outcome (Supplementary File 1c). Taking *SLC12A2* as an example, one standard deviation (SD) decrease in the expression of *SLC12A2* was associated with a decrease of 1.12 (95%CI, 0.80–1.58) mmHg of SBP. It should be noted that the number of SNPs in HEIDI test for gene *P4HA1* was less than five (*n* = 3).

### MR analysis for association of target gene expression in blood and overall and subtypes BC risk

**Fig. 3:**
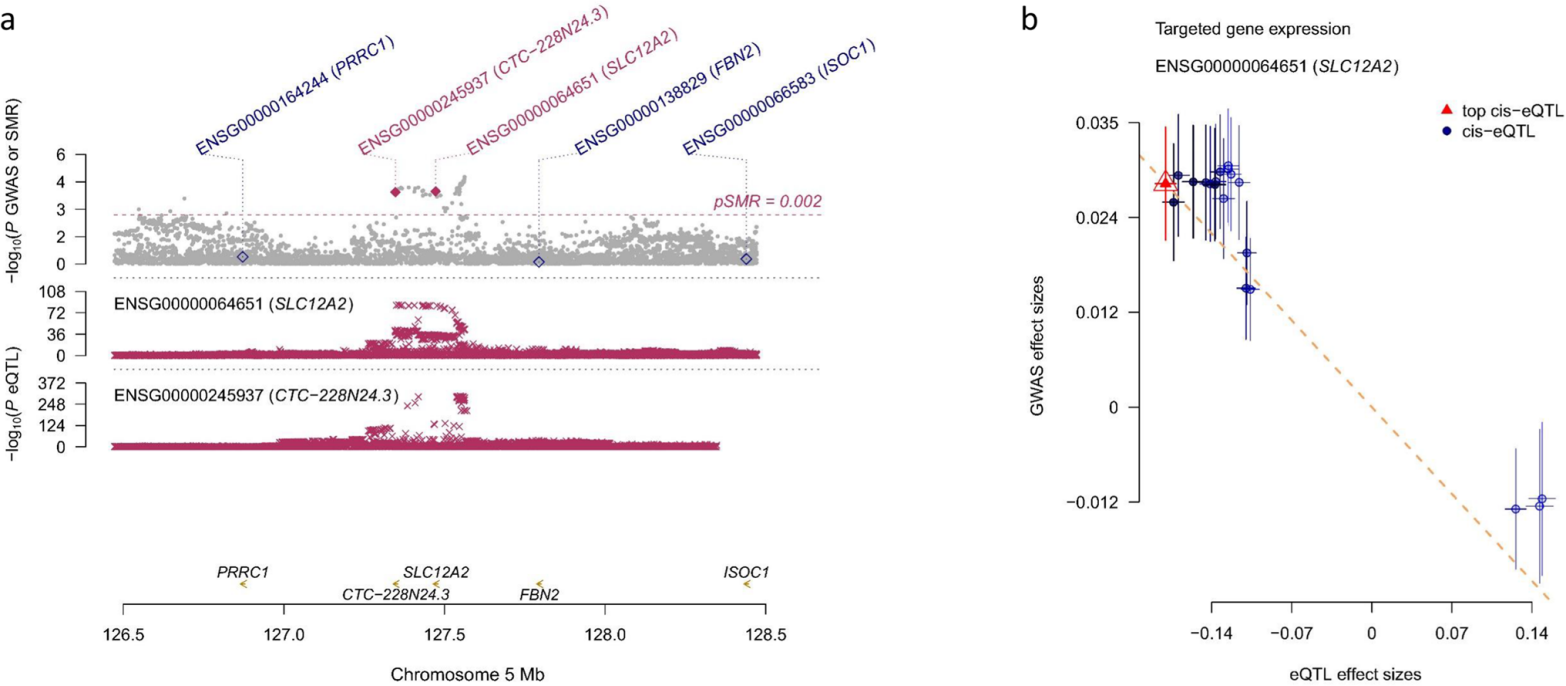
MR association between *SLC12A2* gene expression in blood and breast cancer risk. **a** Top, *p*-values from the GWAS for breast cancer (grey dots) and *p*-values from SMR tests (diamonds). Bottom, *p*-values eQTL data for *SLC12A2* and *CTC-228N24.3*. Shown in a are all the SNPs available in the GWAS and eQTL data. **b** Effect sizes of the SNPs (used for the HEIDI test) from the GWAS against those from the eQTL data. The orange dashed lines represent the estimate of effect size of the MR association at the top cis-eQTL (rather than the regression line). Error bars are the standard errors of SNP effects

In the analysis of MR association between the target gene expressions in blood and overall BC risk, we identified significant associations for five genes including *P4HA1*, *SLC12A2*, *KCNJ11*, *CA12* and *PDE1B*, but only the association for *SLC12A2* was significant after Bonferroni correction (0.05/23 = 2.2 × 10^–3^) (Table 1). Decrease of one SD in the expression of *SLC12A2* was associated with a 16% increase in BC risk (odds ratio, OR, 1.16, 95% confidence interval, 95%CI, 1.06–1.28). This association was complemented by the HEIDI outlier test. There was no substantial difference between the p-value from SMR test compared to that from SMR-multi test. The MR association between *SLC12A2* gene expression in blood and BC risk is shown in Fig. 3. With the same analysis for ER + and ER-BC (Table 2), a nominal significance was observed in the association between ER + BC risk and expressions of *P4HA1*, *SLC12A2*, *KCNJ11* and *PDE1B*, among which *SLC12A2* and *PDE1B* were still significant after Bonferroni correction (0.05/(23 × 2) = 1.1 × 10^–3^). One SD decrease in the *SLC12A2* expression was associated with a 17% increase in the risk of ER+BC (1.17, 1.06–1.28), while one SD decrease in *PDE1B* expression was associated with a 7% decreased risk (0.93, 0.90–0.97). For ER- BC, only *SLC12A2* showed a nominal significance but it was not significant while considering multiple testing. For the molecular subtype of BC (see Table 2), we found *P4HA1*, *SLC12A2*, *KCNJ11* and *CA12* were associated with luminal A-like BC with nominal significance, *P4HA1*, *SLC12A2*, *ATP1A1*, *NR3C1*, *CA12*, *CACNA1D* and *GPR35* for luminal B-like BC, *SLC12A2* and *SCNN1D* for luminal B/HER2-negative-like BC, ACE for HER2-enriched-like BC and AOC1 for triple-negative BC. However, none of them was significant after Bonferroni correction (0.05/(23 × 5) = 4.3 × 10^–4^).

**Table 1:** MR association between drug target gene expression in blood and overall risk of breast cancer

Gene	ProbeChr	Probe_bp	topSNP	topSNP_chr	topSNP_bp	Effect_allele	Other_allele	Freq_Effect_allele	eQTL association			BC association			MR association				HEIDI Test	
									Beta	SE	*p*	Beta	SE	*P*	Beta	SE	*p*	Multi-*p*	*p*	NSNP
SLC12A2	5	127,472,419	rs17764730	5	127,357,526	T	C	0.21	− 0.186	0.009	3.2E–86	0.028	0.007	7.57E–05	− 0.150	0.039	1.05E–04	2.58E–02	1.56E–01	20
P4HA1	10	74,811,853	rs6480668	10	74,849,326	G	A	0.24	0.183	0.016	3.35E–32	− 0.032	0.012	7.83E–03	− 0.176	0.068	9.47E–03	1.30E–03	4.98E–01	8
CA12	15	63,643,968	rs12909041	15	63,743,677	A	C	0.36	0.039	0.010	1.14E–04	− 0.026	0.008	6.88E–04	− 0.670	0.263	1.08E–02	7.12E–01	5.35E–01	5
PDE1B	12	54,958,078	rs10747699	12	54,955,324	T	C	0.39	0.406	0.009	0.00E+00	0.017	0.007	1.31E–02	0.041	0.017	1.32E–02	2.02E–03	3.63E–01	3
KCNJ11	11	17,409,142	rs2074310	11	17,421,886	T	C	0.26	0.157	0.012	4.37E–38	− 0.015	0.006	1.99E–02	− 0.092	0.040	2.20E–02	4.89E–01	8.06E–02	20
NR3C1	5	142,736,286	rs4912908	5	142,791,133	G	A	0.17	0.073	0.010	1.07E–13	0.016	0.008	4.67E–02	0.221	0.115	5.47E–02	3.22E–03	2.81E–02	15
ATP1A1	1	116,934,086	rs6704439	1	116,867,616	A	C	0.30	0.041	0.009	4.39E–06	0.015	0.008	5.47E–02	0.363	0.205	7.64E–02	1.74E–01	8.28E–01	20
KCNJ2	17	68,170,501	rs9890133	17	68,169,005	G	A	0.15	− 0.256	0.012	8.02E–94	− 0.015	0.010	1.12E–01	0.060	0.038	1.13E–01	7.26E–01	9.60E–01	20
SLC12A1	15	48,540,068	rs964611	15	48,597,514	A	C	0.15	1.664	0.009	0.00E+00	− 0.012	0.009	1.78E–01	− 0.007	0.005	1.78E–01	1.21E–01	9.86E–01	20
CACNA2D2	3	50,470,954	rs62260815	3	50,474,624	A	G	0.05	− 0.142	0.013	8.98E–29	− 0.013	0.010	2.03E–01	0.089	0.070	2.06E–01	1.36E–03	3.88E–01	20
AHR	7	17,362,011	rs17643734	7	17,162,882	G	A	0.03	0.478	0.016	3.7E–184	0.015	0.013	2.70E–01	0.030	0.028	2.71E–01	1.18E–02	9.83E–01	20
ACE	17	61,576,813	rs4277405	17	61,548,918	C	T	0.35	0.075	0.009	1.59E–17	0.006	0.006	3.18E–01	0.083	0.084	3.21E–01	9.76E–01	9.97E–01	20
AQP1	7	30,929,070	rs2075574	7	30,950,744	T	C	0.37	0.113	0.013	3.77E–19	− 0.008	0.009	3.50E–01	− 0.073	0.078	3.53E–01	9.92E–01	7.67E–01	5
SLC9A1	1	27,459,389	rs6683016	1	27,493,194	T	C	0.28	− 0.096	0.008	9E–31	− 0.004	0.006	5.37E–01	0.041	0.066	5.38E–01	4.95E–02	7.61E–02	20
AOC1	7	150,540,153	rs7806458	7	150,476,888	G	A	0.28	0.669	0.008	0.00E+00	− 0.003	0.006	6.20E–01	− 0.005	0.009	6.20E–01	4.21E–02	7.63E–01	20
CACNA1H	16	1,237,506	rs117177120	16	1,238,875	G	T	0.02	0.372	0.024	4.41E–54	0.004	0.016	7.82E–01	0.012	0.042	7.82E–01	9.90E–01	4.21E–01	20
SLC16A1	1	113,477,052	rs1049434	1	113,456,546	A	T	0.32	0.125	0.008	9.29E–54	0.002	0.006	8.02E–01	0.013	0.050	8.02E–01	7.22E–01	4.06E–01	20
GPR35	2	241,557,762	rs2975788	2	241,571,858	G	A	0.43	0.293	0.009	6.2E–259	0.002	0.007	8.10E–01	0.005	0.022	8.10E–01	8.83E–01	5.79E–01	20
CACNA1D	3	53,687,586	rs9830632	3	53,735,766	G	A	0.29	− 0.085	0.009	2E–22	− 0.001	0.007	9.19E–01	0.008	0.080	9.19E–01	9.83E–02	3.50E–01	20
ADRB2	5	148,207,176	rs2082395	5	148,200,600	A	G	0.35	0.126	0.008	6.31E–56	− 0.001	0.006	9.37E–01	− 0.004	0.050	9.37E–01	1.02E–01	7.18E–01	4
CA4	17	58,237,778	rs34820870	17	58,244,021	G	T	0.16	− 0.462	0.021	6.1E–110	0.001	0.016	9.50E–01	− 0.002	0.036	9.50E–01	4.38E–02	9.89E–01	20
JUN	1	59,248,125	rs2716140	1	59,472,397	C	A	0.39	− 0.228	0.008	3.5E–177	0.000	0.007	9.57E–01	0.002	0.030	9.57E–01	3.69E–01	9.06E–01	20
SCNN1D	1	1,221,612	rs6662635	1	1,220,425	G	A	0.08	− 0.475	0.044	4.76E–27	− 0.001	0.031	9.80E–01	0.002	0.064	9.80E–01	7.26E–01	9.46E–01	20

Beta of the eQTL association is the standard deviation change in gene expression per coded allele. Beta of the BC association is the log odds per coded allele. Beta of MR association represents the log odds per one standard deviation increase in gene expression. A significant HEIDI *p*-value (< 0.01) indicates that any association between gene expression and outcome may be due to linkage where there are two distinct causal variants in linkage disequilibrium

*MR* Mendelian randomization, *SNP* single nuclear polymorphism, *eQTL* expression quantitative trait loci, *SE* standard error, *BC* breast cancer, *multi-p* *p*-value for SMR estimated based on multiple SNPs, *HEIDI* heterogeneity in dependent instruments, *NSNP* number of SNPs for HEIDI test

**Table 2:** MR association between drug target gene expression in blood and risk of breast cancer subtype according to ER status and molecular characteristics

BC type		Gene	ProbeChr	Probe_bp	topSNP	topSNP_chr	topSNP_bp	Effect_allele	Other_allele	Freq_Effect_allele	eQTL association			BC association			MR association				HEIDI test	
											Beta	SE	*p*	Beta	SE	*p*	Beta	SE	*p*	Multi-*p*	*p*	NSNP
Estrogen receptor status	Positive	P4HA1	10	74,811,853	rs6480668	10	74,849,326	G	A	0.24	0.183	0.016	3.35E–32	− 0.045	0.015	2.49E–03	− 0.247	0.084	3.30E–03	2.02E–04	3.72E–01	8
		SLC12A2	5	127,472,419	rs17764730	5	127,357,526	T	C	0.21	− 0.186	0.009	3.20E–86	0.029	0.009	6.96E–04	− 0.158	0.048	8.67E–04	8.87E–02	1.23E–01	20
		KCNJ11	11	17,409,142	rs2074310	11	17,421,886	T	C	0.26	0.157	0.012	4.37E–38	− 0.016	0.008	4.20E–02	− 0.099	0.050	4.53E–02	5.34E–01	4.33E–01	20
		PDE1B	12	54,958,078	rs1874308	12	54,954,356	A	T	0.39	0.429	0.009	0.00E+00	0.030	0.008	3.38E–04	0.070	0.019	3.28E–04	1.55E–02	2.37E–01	14
	Negative	SLC12A2	5	127,472,419	rs17764730	5	127,357,526	T	C	0.21	− 0.186	0.009	3.20E–86	0.037	0.013	5.13E–03	− 0.200	0.072	5.75E–03	2.21E–01	7.17E–01	20
Molecular characteristics	Luminal A-like	P4HA1	10	74,811,853	rs6480668	10	74,849,326	G	A	0.24	0.183	0.016	3.35E–32	− 0.044	0.016	6.35E–03	− 0.238	0.090	7.84E–03	8.79E–04	9.37E–01	8
		PDE1B	12	54,958,078	rs1874308	12	54,954,356	A	T	0.39	0.429	0.009	0.00E+00	0.023	0.009	9.75E–03	0.053	0.020	9.86E–03	1.66E–02	5.67E–01	14
		CA12	15	63,643,968	rs12909041	15	63,743,677	A	C	0.36	0.039	0.010	1.14E–04	− 0.024	0.010	1.51E–02	− 0.632	0.307	3.97E–02	5.73E–01	5.06E–01	6
		SLC12A2	5	127,472,419	rs17764730	5	127,357,526	T	C	0.21	− 0.186	0.009	3.20E–86	0.019	0.009	4.35E–02	− 0.101	0.050	4.46E–02	8.34E–01	9.57E–01	20
	Luminal B-like	P4HA1	10	74,811,853	rs6480668	10	74,849,326	G	A	0.24	0.183	0.016	3.35E–32	− 0.093	0.037	1.11E–02	− 0.506	0.204	1.31E–02	3.38E–02	9.98E–01	8
		GPR35	2	241,557,762	rs2975788	2	241,571,858	G	A	0.43	0.293	0.009	6.23E–259	− 0.044	0.019	2.02E–02	− 0.151	0.065	2.05E–02	4.70E–01	1.21E–02	20
		SLC12A2	5	127,472,419	rs17764730	5	127,357,526	T	C	0.21	− 0.186	0.009	3.20E–86	0.044	0.020	3.24E–02	− 0.236	0.111	3.34E–02	2.45E–02	1.73E–02	20
		CACNA1D	3	53,687,586	rs9830632	3	53,735,766	G	A	0.29	− 0.085	0.009	2.00E–22	0.042	0.020	3.17E–02	− 0.499	0.238	3.59E–02	1.59E–01	6.17E–01	20
		CA12	15	63,643,968	rs12909041	15	63,743,677	A	C	0.36	0.039	0.010	1.14E–04	− 0.056	0.023	1.29E–02	− 1.457	0.697	3.66E–02	9.74E–01	8.70E–01	6
		ATP1A1	1	116,934,086	rs6704439	1	116,867,616	A	C	0.30	0.041	0.009	4.39E–06	0.050	0.022	2.10E–02	1.216	0.590	3.92E–02	3.08E–01	9.63E–01	20
		NR3C1	5	142,736,286	rs4912908	5	142,791,133	G	A	0.17	0.073	0.010	1.07E–13	0.047	0.023	3.76E–02	0.649	0.324	4.53E–02	1.80E–01	3.31E–01	14
	luminal B/HER2-negativE–like	SLC12A2	5	127,472,419	rs17764730	5	127,357,526	T	C	0.21	− 0.186	0.009	3.20E–86	0.054	0.017	1.82E–03	− 0.292	0.095	2.08E–03	4.36E–02	6.53E–01	20
		SCNN1D	1	1,221,612	rs6662635	1	1,220,425	G	A	0.08	− 0.475	0.044	4.76E–27	− 0.206	0.080	1.03E–02	0.434	0.174	1.26E–02	7.41E–01	3.35E–01	20
	HER2-enriched	KCNJ11	11	17,409,142	rs2074310	11	17,421,886	T	C	0.26	0.157	0.012	4.37E–38	− 0.041	0.027	1.28E–01	− 0.258	0.171	1.31E–01	5.89E–01	6.21E–01	20
		ACE	17	61,576,813	rs4277405	17	61,548,918	C	T	0.35	0.075	0.009	1.59E–17	0.069	0.026	7.99E–03	0.918	0.363	1.13E–02	7.14E–01	1.93E–01	20
	TriplE–negative	AOC1	7	150,540,153	rs7806458	7	150,476,888	G	A	0.28	0.669	0.008	0.00E+00	− 0.031	0.016	4.97E–02	− 0.046	0.024	4.97E–02	2.68E–01	9.62E–01	20

Beta of the eQTL association is the standard deviation change in gene expression per coded allele. Beta of the BC association is the log odds per coded allele. Beta of MR association represents the log odds per one standard deviation increase in gene expression. A significant HEIDI *p*-value (< 0.01) indicates that any association between gene expression and outcome may be due to linkage where there are two distinct causal variants in linkage disequilibrium

*MR* Mendelian randomization, *SNP* single nuclear polymorphism, *eQTL* expression quantitative trait loci, *SE* standard error, *BC* breast cancer, *multi-p* *p*-value for SMR estimated based on multiple SNPs, *HEIDI* heterogeneity in dependent instruments, *NSNP* number of SNPs for HEIDI test

### Sensitivity analyses

We conducted several sensitivity analyses to evaluate the discovered significant associations between targetable gene expression and BC risk. The associations for the top SNP pertaining to *SLC12A2* and *PDE1B* with nearby genes within the 2 MB window are shown in Supplementary File 1d. In the test for horizontal pleiotropy, gene *CTC-228N24.3* within the 2 MB window of *SLC12A2* was also found significantly associated with BC risk (Supplementary File 1e). The p-value of the SMR association for *CTC-228N24.3* was similar to that of *SLC12A2*. However, the posterior probability for a common variant between *CTC-228N24.3* and overall BC risk was 77%, less than that for *SLC12A2* (81.5%). The posterior probability for a common causal variant between *SLC12A2* expression and risk of ER + BC was 40.5% and for *PDE1B* was 66.8%. None of the corresponding nearby genes showed a higher probability than *SLC12A2* and *PDE1B*.

**Fig. 4:**
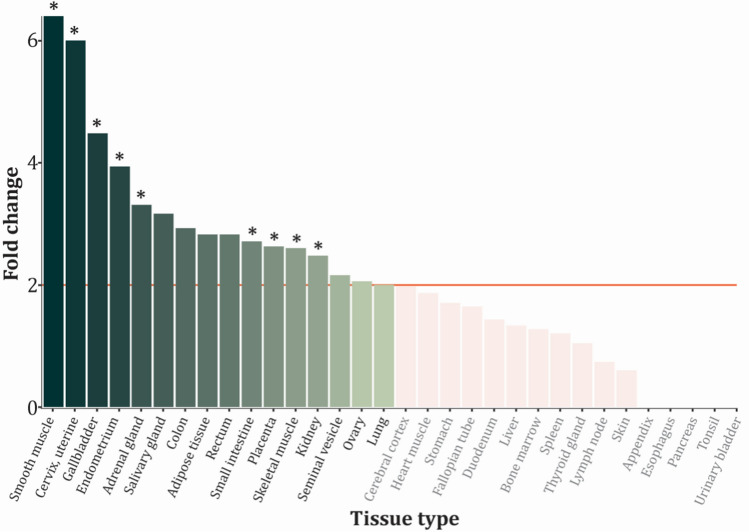
Tissue enrichment identifies tissue types where the target genes are most likely to be differentially expressed. Color gradient represents decreasing fold change and light reds are insignificant associations. Stars indicate statistical significance in the tests

In the tissue enrichment analysis, 16 different tissues were found enriched with the collective targeted genes (Fig. 4). We further measured the statistical significance of the associations which largely overlapped with the observation of fold change. Here target genes were found to be enriched in several tissues with fold change over 2 (smooth muscle, cervix, gallbladder, endometrium, adrenal gland, small intestine, placenta, skeletal muscle, and kidney). The MR associations for all available tissue are shown in Supplementary File 1f. We identified several genes in adrenal gland, mammary tissue, kidney cortex and uterus that were associated with BC risk, but none of them were significant after considering Bonferroni correction (0.05/(23 × 7) = 3.1 × 10^–4^).

We identified five data resources in Expression Atlas (Supplementary File 1 g), which contained gene expression of *SLC12A2* for invasive BC tissue (or other tissue from BC patients) and normal tissue (or other tissue from healthy controls). The samples were either from blood or breast tissue. In three datasets, *SLC12A2* expression in BC tissue was found significantly lower compared to that in normal breast tissue from BC patients. Another dataset indicated *SLC12A2* expression in blood platelet from patients with breast carcinoma was lower than that from healthy controls (log fold change = − 3.3, *p*-value = 7.7 × 10^–24^). Only one showed the opposite association where the type of the BC was invasive ductal carcinoma (log fold change = 2.5, p-value = 0.0286).

The phenotypes that were associated with the top SNP for *SLC12A2* gene expression are listed in Supplementary File 1 h. Interestingly, most of the traits were related to obesity.

We then performed MR with the GSMR method as well as with TwoSampleMR to test if SBP was in fact associated with overall BC risk. The results largely indicated no evidence of the causal association between genetically estimated SBP and BC risk (Supplementary File 1i).

## Discussions

With this two-sample MR study, we observed that the overall BC risk was increased 16% along with one SD decrease of *SLC12A2* gene expression and shared a similar effect with its divergent transcript *CTC-228N24.3*. *SLC12A2* encodes Na(+)-K(+)-2Cl(–) co-transporter isoform 1 (NKCC1), which can be targeted by loop diuretics including torasemide, bumetanide and quinethazone. Thus, long-term use of these drugs may potentially increase overall BC risk. The significant association with the expression of *SLC12A2* was further observed with ER+BC. In addition, a SD decrease in expression of *PDE1B*, which can be targeted by three CCBs: nicardipine, felodipine and bepridil, was associated with 7% decreased risk of ER+BC. We did not observe any significant association between other target gene expressions and BC risk.

A recent meta-analysis evaluated the association between antihypertensive medication use and BC according to 57 conventional observational studies and also reported that use of diuretics was associated with increased BC risk [28]. However, this study also found an increased BC risk among users of BBs, or CCBs [28], which is inconsistent with our result. Another meta-analysis based on four studies found that the relative risk of BC among users of loop diuretics was 0.91 (0.77–1.06) compared to nonusers, in possible disagreement with our findings [29]. A RCT-based meta-analysis concluded no consistent evidence that antihypertensive medication use had any effect on cancer risk [3]. The inconsistent findings may be due to the difference in study population, exposure assessment and the control of confounding effects. The observational studies are subjected to selection bias, information bias, and confounding by indication. Our results are in agreement with the findings from Yarmolinsky et al. who investigated antihypertensive drug use and risk of common cancers by evaluating SNPs in *ACE*, *ADRB1*, and *SLC12A3* in GWAS of SBP to proxy genetic inhibition of the angiotensin-converting enzyme (ACE), β-1 adrenergic receptor (ADRB1), and sodium-chloride symporter (NCC) [13]. None of the inhibited targets were associated with the risk of BC [13]. Unlike this MR study which only considered one target for each type of antihypertensive drug, we included all the potential targets.

The mechanisms that links *SLC12A2* gene expression and BC risk are unclear. The GSMR analysis for the association between SBP and BC risk indicates that the effect of the drugs on BC risk was not through blood pressure, which is consistent with the finding that there is no evidence of genetic correlation between blood pressure and BC risk [30]. The *SLC12A2*-coded protein, NKCC1 has been reported to be involved in mammary gland development and regulate breast morphogenesis [31]. So far, very few studies have investigated the association of BC risk and use of loop diuretics, although among which furosemide targets also NKCC2, a homogeneous protein of NKCC1. Elsewhere an in vitro study reported expression of *SLC12A2* in BC cells to be downregulated by the treatment with estrogen [32], which is a well-known risk factor for BC. Our identification of phenotypes related to the top SNP of *SLC12A2* indicates obesity may contribute to the increased BC risk linked to *SLC12A2* gene expression. Interestingly, loss of SLC12A2 in pancreatic β-cells was associated with weight gain in mice [33]. Obesity was recognized as a risk factor for particularly ER+BC [34]. This is in accordance with our current study where *SLC12A2* expression was found protective against BC risk and this association was only significant for ER+BC. It is incumbent on further studies to investigate if *SLC12A2* gene expression affects BC risk through obesity/estrogen-related pathways. It should be noted that the case number of ER-BC GWAS was much less than ER+BC GWAS which might detail the loss of signal. Four out of five datasets from Expression Atlas also indicated lower expression of *SLC12A2* in BC tissue (or tissue from BC patients) than the normal tissue (or tissue from controls). On contrary, several in vitro studies reported that inhibition of NKCC1 could reduce cell proliferation, invasion and/or migration in glioblastoma, glioma, esophageal squamous cell carcinoma, hepatocellular carcinoma and gastric cancer cells which may indicate a global underlying tumor-inhibiting mechanism [35–41]. Sun et al. also found that increased expression of NKCC1 was associated with poor prognosis in lung adenocarcinoma and EGFR-mutated adenocarcinoma [42]. More studies are required to investigate the role of NKCC1 in BC etiology.

In our sensitivity analysis, we found that the expression of the divergent transcript of *SCL12A2*, *CTC-228N24.3* was also associated with BC risk. It is non-trivial to decouple the two genes merely based on the summary statistics as they largely belong to the same haplogroup. In previous GWAS both genes have been implicated for BC (study ID in GWAS Catalog: GCST004988) and for blood pressure (GCST90132903-05 and GCST90020232-37), respectively, often via rs17764730 or other SNPs in LD such as rs1112956 and rs36715. As the eQTL results point out, the rs17764730 SNP has tangible cis-effects on both of them and our analyses do not discount one over the other, rather speculate on possible mechanisms between the protein coding gene of the two having a causal effect on BC risk. *CTC-228N24.3*, a lincRNA encoder (RNA1184), also known as ENSG00000245937 is a divergent transcript to *SLC12A2* and mutually share enhancer/promoters GH05J128081 and GH05J128534 (GeneHancer data) where the rs17764730 resides. Previous studies showed mutation in this region regulate expression modulation of both genes [43]. Our observations may only provide a plausible biological basis for the conclusions drawn, which remain yet to be confirmed with further functional investigations.

*PDE1B* was the only other gene that showed the association with ER+BC risk. The function of protein PDE1B includes dopaminergic signaling, immune cell activation, and cell survival. It is one of the family members of phosphodiesterases (PDEs) that hydrolyze cyclic adenosine monophosphate (cAMP) and cyclic guanosine monophosphate (cGMP); the latter two participate in many physiological processes such as visual transduction, cell proliferation and differentiation, and cell-cycle regulation [44]. Some tumor cells overexpress PDEs and as a consequence, the level of cAMP/cGMP in tumor cells is lower than the normal cells [45]. PDEs have become the potential therapeutic target to increase intracellular cAMP/cGMP and thus inhibit tumor growth [46, 47]. PDE1B can be targeted by miR-5701 to inhibit proliferation and promote apoptosis of clear cell renal cell carcinoma cells [48]. Wittliff et al. used gene expression of *PDE1B* and several other genes to predict the overall survival of breast carcinoma [49]. However, in the tissue-specific MR, the results for the mammary gland contradicted the current evidence.

We need to emphasize the strengths and weaknesses of this study. Use of GWAS summary statistics with two sample MR increased statistical power. The application of genetic variants to proxy the use of antihypertensive medication reduced the chance of confounding, misclassification and immortal time bias that are common caveats in a conventional observational study [50]. The inherited variants were present since conception affecting the target gene expression, which provided the opportunity to observe the effect of antihypertensive medication on BC risk in the long term. Compared to RCT, our study design is more efficient as cancer is a late-onset disorder and needs long-term follow-up. Using a plethora of publicly available summary statistics data from GWAS, MR was performed without recruiting new patients or designing additional studies like RCT. We conducted multiple sensitivity analyses such as colocalization analysis and pleiotropy test to evaluate the viability of the assumptions of the instrument variables and to reduce chance findings. As for limitations, the exploration of the molecular subtype of BC was underpowered due to small sample size. Larger BC subtype-specific GWAS data from the consortium are needed to remedy such issue. Low statistical power was also a limitation for the localization analyses. In addition, the coloc package assumes a single causal variant for both traits in the genomic region examined, but multiple conditionally independent variants may exist in regions while ‘coloc.SuSiE’ was unable to provide credible set for BC GWAS. We were unable to exclusively identify the causal effect from *SLC12A2* and *SLC12A2-DT*. Horizontal pleiotropy was assessed via differential expression of other genes, but it is uncertain if there were possible horizontal pleiotropic effects through other related traits. The instrumental variants for the exposure were based on eQTL data derived from both genders and the BC GWAS data were only based on females. Therefore, future studies need to investigate whether the actions of antihypertensive target genes on BC risk are gender specific. The effect of differed gene expression on BC risk due to polymorphism may not be the same as that due to the use of antihypertensive medication, as the former exposure is from early lifetime and the latter mostly from adulthood. Therefore, our results provide a strong signal to select existing drugs (NKCC1-targeted antihypertensive medication) to be validated with RCT. Additionally, our results suggest future studies could focus on the association between expression level of NKCC1 protein and BC risk, which may provide evidence on risk reduction.

## Conclusions

By using two-sample MR, we found BC risk was negatively associated with expression of *SLC12A2,* and ER+BC risk was positively associated with expression of *PDE1B*. Therefore, expression modulation of *SLC12A2* and *PDE1B* via antihypertensive drug usage (in addition to possible impacts through *SLC12A2-DT*) was associated respectively with increased and decreased risk of BC. The observed effect on the BC risk was independent of systolic blood pressure.

### Supplementary Information

Below is the link to the electronic supplementary material.

Supplementary file1 (DOCX 103 kb)

## Data Availability

The data that support the findings of this study are publicly available. The sources have been summarized in Supplementary File 1a.
