# Supplementary material for: Antihypertensive drug targets and breast cancer risk: a two-sample Mendelian randomization study
Source: Eur J Epidemiol. 2024 Feb 24;39(5):535–48. doi: 10.1007/s10654-024-01103-x (PMC11219410; doi:10.1007/s10654-024-01103-x)
Supplement: Supplementary file 1 — Supplementary file1 (DOCX 103 kb) [file 10654_2024_1103_MOESM1_ESM.docx]

**Supplementary File 1a** Data resource used in the current study

| **Category** | **Resource** | **URL** |
| --- | --- | --- |
| List of antihypertensive drugs | WHO Collaborating Centre for Drug Statistics Methodology | <https://www.whocc.no/> |
| Identification of targeted gene | Drugbank | <https://go.drugbank.com/> |
| Genetic instruments for targeted gene expression in blood | eQTLGen Consortium | <https://www.eqtlgen.org/> |
| Systolic blood pressure GWAS | GRASP | <https://grasp.nhlbi.nih.gov/FullResults.aspx> |
| Breast cancer GWAS | Breast Cancer Association Consortium | <https://bcac.ccge.medschl.cam.ac.uk/bcacdata/oncoarray/oncoarray-and-combined-summary-result/> |
| Differential gene expression | Expression Atlas | <https://www.ebi.ac.uk/gxa/home> |
| Traits associated with the top SNP of the target gene expression | PhenoScanner (v2) | <http://www.phenoscanner.medschl.cam.ac.uk/> |

**Supplementary File 1b** Genes targeted by antihypertensive medications in DrugBank

| **Drug Class** | **Drug subclass** | **Medication Subclass** | **ATC Code** | **Drug name** | **Chemical name (IUPAC)** | **Trade name** | **ATC Code** | **Target Gene(s)** |
| --- | --- | --- | --- | --- | --- | --- | --- | --- |
| Other a ntihypertensives | Anti-adrenergic agents, centrally- acting | Rauwolfia alkaloids | C02AA | rescinnamine | methyl (1R,15S,17R,18R,19S,20S)-6,18-dimethoxy-17-{[3-(3,4,5-trimethoxyphenyl)prop-2-enoyl]oxy}-3,13-diazapentacyclo[11.8.0.0^{2,10}.0^{4,9}.0^{15,20}]henicosa-2(10),4(9),5,7-tetraene-19-carboxylate | Moderil, Cinnasil, Anaprel | C02AA01 | ACE |
|  |  |  |  | reserpine | methyl (1R,15S,17R,18R,19S,20S)-6,18-dimethoxy-17-(3,4,5-trimethoxybenzoyloxy)-3,13-diazapentacyclo[11.8.0.0^{2,10}.0^{4,9}.0^{15,20}]henicosa-2(10),4(9),5,7-tetraene-19-carboxylate | Renese-R | C02AA02 | BIRC5, SLC18A1, SLC18A2 |
|  |  |  |  | rauwolfia alkaloids | Not Available |  | C02AA04 | SLC18A2 |
|  |  |  |  | deserpidine | methyl (1R,15S,17R,18R,19S,20S)-18-methoxy-17-(3,4,5-trimethoxybenzoyloxy)-3,13-diazapentacyclo[11.8.0.0^{2,10}.0^{4,9}.0^{15,20}]henicosa-2(10),4,6,8-tetraene-19-carboxylate |  | C02AA05 | SLC18A2 |
|  |  |  |  | methoserpidine | methyl (1R,15S,17R,18R,19S,20S)-7,18-dimethoxy-17-(3,4,5-trimethoxybenzoyloxy)-3,13-diazapentacyclo[11.8.0.0^{2,10}.0^{4,9}.0^{15,20}]henicosa-2(10),4,6,8-tetraene-19-carboxylate |  | C02AA06 |  |
|  |  |  |  | bietaserpine | methyl (1R,15S,17R,18R,19S,20S)-3-[2-(diethylamino)ethyl]-6,18-dimethoxy-17-(3,4,5-trimethoxybenzoyloxy)-3,13-diazapentacyclo[11.8.0.0^{2,10}.0^{4,9}.0^{15,20}]henicosa-2(10),4,6,8-tetraene-19-carboxylate |  | C02AA07 |  |
|  |  | Methyldopa | C02AB | methyldopa | (2S)-2-amino-3-(3,4-dihydroxyphenyl)-2-methylpropanoic acid | Aldomet, Aldoril, Dopamet | C02AB01, C02AB02 | ADRA2A, DDC |
|  |  | Imidazoline receptor agonists | C02AC | clonidine |  | Catapres, Catapres-TTS, Catapres-tts-1, Duraclon, Kapvay, Nexiclon XR | C02AC01 | ADRA2A, ADRA2C, ADRA1A, ADRA2B, ADRA1B, ADRA1D |
|  |  |  |  | guanfacine | N-carbamimidoyl-2-(2,6-dichlorophenyl)acetamide | Intuniv, Tenex | C02AC02 | ADRA2B, ADRA2A |
|  |  |  |  | tolonidine | N-(2-chloro-4-methylphenyl)-4,5-dihydro-1H-imidazol-2-amine |  | C02AC04 |  |
|  |  |  |  | moxonidine | 4-chloro-N-(4,5-dihydro-1H-imidazol-2-yl)-6-methoxy-2-methylpyrimidin-5-amine | Physiotens, Moxon | C02AC05 | ADRA2A, NISCH |
|  |  |  |  | rilmenidine | N-(dicyclopropylmethyl)-4,5-dihydro-1,3-oxazol-2-amine | Albarel, Hyperium, Iterium and Tenaxum | C02AC06 | ADRA2A |
|  | Anti-adrenergic agents, ganglion-blocking | Sulfonium derivatives | C02BA | trimetaphan | 3,5-dibenzyl-4-oxo-8lambda4-thia-3,5-diazatricyclo[6.3.0.0^{2,6}]undecan-8-ylium | Arfonad | C02BA01 | CHRNA10 |
|  |  | Secondary and tertiary amines | C02BB | mecamylamine | N,2,3,3-tetramethylbicyclo[2.2.1]heptan-2-amine | Inversine, Vecamyl | C02BB01 | CHRNA2, CHRNA4, CHRNB2, CHRNA7 |
|  | Anti-adrenergic agents, peripherally- acting | Alpha-adrenoreceptor antagonists | C02CA | prazosin | 2-[4-(furan-2-carbonyl)piperazin-1-yl]-6,7-dimethoxyquinazolin-4-amine | Minipress, Minizide | C02CA01 | ADRA1A, ADRA1B, ADRA1D, KCNH2, ADRA2A, ADRA2B |
|  |  |  |  | indoramin | N-{1-[2-(1H-indol-3-yl)ethyl]piperidin-4-yl}benzamide | Baratol, Doralese | C02CA02 | ADRA1A |
|  |  |  |  | trimazosin | 2-hydroxy-2-methylpropyl 4-(4-amino-6,7,8-trimethoxyquinazolin-2-yl)piperazine-1-carboxylate |  | C02CA03 |  |
|  |  |  |  | doxazosin | 2-[4-(2,3-dihydro-1,4-benzodioxine-2-carbonyl)piperazin-1-yl]-6,7-dimethoxyquinazolin-4-amine | Cardura | C02CA04 | ADRA1A, ADRA1B, ADRA1D, KCNH2, KCNH6, KCNH7 |
|  |  |  |  | urapidil | 6-({3-[4-(2-methoxyphenyl)piperazin-1-yl]propyl}amino)-1,3-dimethyl-1,2,3,4-tetrahydropyrimidine-2,4-dione |  | C02CA06 |  |
|  |  | Guanidine derivatives | C02CC | betanidine | (E)-N'-benzyl-N,N''-dimethylguanidine |  | C02CC01 | KCNJ1, ADRA2A, ADRA2B, ADRA2C, ADRB1, ADRB2, ADRB3 |
|  |  |  |  | guanethidine | N''-[2-(azocan-1-yl)ethyl]guanidine |  | C02CC02 | SLC6A2 |
|  |  |  |  | guanoxan | N-[(2,3-dihydro-1,4-benzodioxin-2-yl)methyl]guanidine |  | C02CC03 |  |
|  |  |  |  | debrisoquine | 1,2,3,4-tetrahydroisoquinoline-2-carboximidamide |  | C02CC04 | SLC6A2 |
|  |  |  |  | guanoclor | N-{[2-(2,6-dichlorophenoxy)ethyl]amino}guanidine |  | C02CC05 |  |
|  |  |  |  | guanazodine | N-[(azocan-2-yl)methyl]guanidine |  | C02CC06 |  |
|  |  |  |  | guanoxabenz | N'-[(E)-[(2,6-dichlorophenyl)methylidene]amino]-N-hydroxyguanidine |  | C02CC07 |  |
|  | arteriolar smooth muscle, agents acting on | Thiazide derivatives | C02DA | diazoxide | 7-chloro-3-methyl-4H-1lambda6,2,4-benzothiadiazine-1,1-dione | Proglycem | C02DA01 | CA1, CA2, KCNMA1, KCNJ11, ATP1A1 |
|  |  | Hydrazinophthalazine derivatives | C02DB | dihydralazine | 1,4-dihydrazinylphthalazine |  | C02DB01 |  |
|  |  |  |  | hydralazine | 1-hydrazinylphthalazine | Apresoline, Bidil | C02DB02 | P4HA1, AOC3, HIF1A |
|  |  |  |  | endralazine | (3Z)-6-benzoyl-3-hydrazinylidene-2H,3H,5H,6H,7H,8H-pyrido[4,3-c]pyridazine |  | C02DB03 |  |
|  |  |  |  | cadralazine | N'-{6-[ethyl(2-hydroxypropyl)amino]pyridazin-3-yl}ethoxycarbohydrazide |  | C02DB04 |  |
|  |  | Pyrimidine derivatives | C02DC | minoxidil | 2,6-diamino-4-(piperidin-1-yl)pyrimidin-1-ium-1-olate | Loniten, Minox, Regoxidine, Rogaine | C02DC01 | PTGS1, REN, KCNJ1 |
|  |  | Nitroferricyanide derivatives | C02DD | nitroprusside | pentacyano(nitroso)irondiuide | Nipride, Nipride RTU, Nitropress | C02DD01 | NPR1 |
|  |  | Guanidine derivatives | C02DG | pinacidil | (Z)-N'-cyano-N-(3,3-dimethylbutan-2-yl)-N''-(pyridin-4-yl)guanidine |  | C02DG01 |  |
|  | other antihypertensives | Alkaloids, excl. rauwolfia | C02KA | veratrum |  |  | C02KA01 |  |
|  |  | Tyrosine hydroxylase inhibitors | C02KB | metirosine | (2S)-2-amino-3-(4-hydroxyphenyl)-2-methylpropanoic acid | Demser | C02KB01 | TH |
|  |  | MAO inhibitors | C02KC | pargyline | benzyl(methyl)(prop-2-yn-1-yl)amine | Eutonyl | C02KC01 | MAOB, MAOA |
|  |  | Serotonin antagonists | C02KD | ketanserin | 3-{2-[4-(4-fluorobenzoyl)piperidin-1-yl]ethyl}-1,2,3,4-tetrahydroquinazoline-2,4-dione | Sufrexal | C02KD01 | HTR2A |
|  |  | Antihypertensives for pulmonary arterial hypertension | C02KX | bosentan | 4-tert-butyl-N-[6-(2-hydroxyethoxy)-5-(2-methoxyphenoxy)-[2,2'-bipyrimidin]-4-yl]benzene-1-sulfonamide | Stayveer, Tracleer | C02KX01 | EDNRB, EDNRA |
|  |  |  |  | ambrisentan | (2S)-2-[(4,6-dimethylpyrimidin-2-yl)oxy]-3-methoxy-3,3-diphenylpropanoic acid | Letairis | C02KX02 | EDNRB, EDNRA |
|  |  |  |  | sitaxentan | N-(4-chloro-3-methyl-1,2-oxazol-5-yl)-2-[2-(6-methyl-2H-1,3-benzodioxol-5-yl)acetyl]thiophene-3-sulfonamide |  | C02KX03 | EDNRB, EDNRA |
|  |  |  |  | macitentan | {[5-(4-bromophenyl)-6-{2-[(5-bromopyrimidin-2-yl)oxy]ethoxy}pyrimidin-4-yl]sulfamoyl}(propyl)amine | Opsumit | C02KX04 | EDNRB, EDNRA |
|  |  |  |  | riociguat | methyl N-(4,6-diamino-2-{1-[(2-fluorophenyl)methyl]-1H-pyrazolo[3,4-b]pyridin-3-yl}pyrimidin-5-yl)-N-methylcarbamate | Adempas | C02KX05 | GUCY1A2 |
| ACEi | ACE inhibitors, plain | ACE inhibitors, plain | C09AA | captopril | (2S)-1-[(2S)-2-methyl-3-sulfanylpropanoyl]pyrrolidine-2-carboxylic acid | Capoten | C09AA01 | MMP9, ACE, MMP2, LTA4H, BDKRB1 |
|  |  |  |  | enalapril | (2S)-1-[(2S)-2-{[(2S)-1-ethoxy-1-oxo-4-phenylbutan-2-yl]amino}propanoyl]pyrrolidine-2-carboxylic acid | Epaned, Vaseretic, Vasotec | C09AA02 | ACE |
|  |  |  |  | lisinopril | (2S)-1-[(2S)-6-amino-2-{[(1S)-1-carboxy-3-phenylpropyl]amino}hexanoyl]pyrrolidine-2-carboxylic acid | Prinivil, Qbrelis, Zestoretic, Zestril | C09AA03 | ACE, REN |
|  |  |  |  | perindopril | (2S,3aS,7aS)-1-[(2S)-2-{[(2S)-1-ethoxy-1-oxopentan-2-yl]amino}propanoyl]-octahydro-1H-indole-2-carboxylic acid | Aceon, Coversyl, Prestalia, Viacoram | C09AA04 | ACE, SFRP4 |
|  |  |  |  | ramipril | (2S,3aS,6aS)-1-[(2S)-2-{[(2S)-1-ethoxy-1-oxo-4-phenylbutan-2-yl]amino}propanoyl]-octahydrocyclopenta[b]pyrrole-2-carboxylic acid | Altace, Altace HCT | C09AA05 | ACE, BDKRB1 |
|  |  |  |  | quinapril | (3S)-2-[(2S)-2-{[(2S)-1-ethoxy-1-oxo-4-phenylbutan-2-yl]amino}propanoyl]-1,2,3,4-tetrahydroisoquinoline-3-carboxylic acid | Accupril, Accuretic | C09AA06 | ACE |
|  |  |  |  | benazepril | 2-[(3S)-3-{[(2S)-1-ethoxy-1-oxo-4-phenylbutan-2-yl]amino}-2-oxo-2,3,4,5-tetrahydro-1H-1-benzazepin-1-yl]acetic acid | Amlobenz, Lotensin, Lotensin Hct, Lotrel | C09AA07 | ACE |
|  |  |  |  | cilazapril | (1S,9S)-9-{[(2S)-1-ethoxy-1-oxo-4-phenylbutan-2-yl]amino}-10-oxo-octahydro-1H-pyridazino[1,2-a][1,2]diazepine-1-carboxylic acid | Inhibace | C09AA08 | ACE |
|  |  |  |  | fosinopril | (2S,4S)-4-cyclohexyl-1-(2-{[(1S)-2-methyl-1-(propanoyloxy)propoxy](4-phenylbutyl)phosphoryl}acetyl)pyrrolidine-2-carboxylic acid | Monopril | C09AA09 | ACE |
|  |  |  |  | trandolapril | (2S,3aR,7aS)-1-[(2S)-2-{[(2S)-1-ethoxy-1-oxo-4-phenylbutan-2-yl]amino}propanoyl]-octahydro-1H-indole-2-carboxylic acid | Mavik, Tarka | C09AA10 | ACE |
|  |  |  |  | spirapril | (8S)-7-[(2S)-2-{[(2S)-1-ethoxy-1-oxo-4-phenylbutan-2-yl]amino}propanoyl]-1,4-dithia-7-azaspiro[4.4]nonane-8-carboxylic acid | Renormax | C09AA11 | ACE |
|  |  |  |  | delapril | 2-[(2S)-N-(2,3-dihydro-1H-inden-2-yl)-2-{[(2S)-1-ethoxy-1-oxo-4-phenylbutan-2-yl]amino}propanamido]acetic acid |  | C09AA12 |  |
|  |  |  |  | moexipril | (3S)-2-[(2S)-2-{[(2S)-1-ethoxy-1-oxo-4-phenylbutan-2-yl]amino}propanoyl]-6,7-dimethoxy-1,2,3,4-tetrahydroisoquinoline-3-carboxylic acid | Univasc | C09AA13 | ACE |
|  |  |  |  | temocapril | InChI=1S/C23H28N2O5S2/c1-2-30-23(29)17(11-10-16-7-4-3-5-8-16)24-18-15-32-20(19-9-6-12-31-19)13-25(22(18)28)14-21(26)27/h3-9,12,17-18,20,24H,2,10-11,13-15H2,1H3,(H,26,27)/t17-,18-,20-/m0/s1 | Acecol | C09AA14 | ACE |
|  |  |  |  | zofenopril | (2S,4S)-1-[(2S)-3-(benzoylsulfanyl)-2-methylpropanoyl]-4-(phenylsulfanyl)pyrrolidine-2-carboxylic acid | Zocardis | C09AA15 | ACE |
|  |  |  |  | imidapril | (4S)-3-[(2S)-2-{[(2S)-1-ethoxy-1-oxo-4-phenylbutan-2-yl]amino}propanoyl]-1-methyl-2-oxoimidazolidine-4-carboxylic acid | Tanatril | C09AA16 |  |
| ARBs | Angiotensin ii receptor blockers (ARBs), plain | Angiotensin II receptor blockers (ARBs), plain | C09CA | losartan | (2-butyl-4-chloro-1-{[2'-(2H-1,2,3,4-tetrazol-5-yl)-[1,1'-biphenyl]-4-yl]methyl}-1H-imidazol-5-yl)methanol | Cozaar, Hyzaar | C09CA01 | AGTR1 |
|  |  |  |  | eprosartan | 4-({2-butyl-5-[(1E)-2-carboxy-2-[(thiophen-2-yl)methyl]eth-1-en-1-yl]-1H-imidazol-1-yl}methyl)benzoic acid | Teveten HCT | C09CA02 | AGTR2 |
|  |  |  |  | valsartan | (2S)-3-methyl-2-(N-{[2'-(2H-1,2,3,4-tetrazol-5-yl)-[1,1'-biphenyl]-4-yl]methyl}pentanamido)butanoic acid | Dafiro, Diovan, Diovan Hct, Entresto, Exforge, Exforge Hct | C09CA03 | AGTR3 |
|  |  |  |  | irbesartan | 2-butyl-3-{[2'-(2H-1,2,3,4-tetrazol-5-yl)-[1,1'-biphenyl]-4-yl]methyl}-1,3-diazaspiro[4.4]non-1-en-4-one | Avalide, Avapro, Ifirmacombi, Karvea, Karvezide | C09CA04 | AGTR1, JUN |
|  |  |  |  | tasosartan | 2,4-dimethyl-8-{[2'-(2H-1,2,3,4-tetrazol-5-yl)-[1,1'-biphenyl]-4-yl]methyl}-5H,6H,7H,8H-pyrido[2,3-d]pyrimidin-7-one |  | C09CA05 | AGTR1, AGTR2 |
|  |  |  |  | candesartan | 2-ethoxy-1-{[2'-(1H-1,2,3,4-tetrazol-5-yl)-[1,1'-biphenyl]-4-yl]methyl}-1H-1,3-benzodiazole-7-carboxylic acid | Atacand Plus, Hytacand, Blopress Plus, Advantec and Ratacand Plus | C09CA06 | AGTR1 |
|  |  |  |  | telmisartan | 4'-{[4-methyl-6-(1-methyl-1H-1,3-benzodiazol-2-yl)-2-propyl-1H-1,3-benzodiazol-1-yl]methyl}-[1,1'-biphenyl]-2-carboxylic acid | Actelsar Hct, Micardis, Micardis-hct, Pritor, Twynsta | C09CA07 | AGTR1, PPARG |
|  |  |  |  | olmesartan medoxomil | 4-(2-hydroxypropan-2-yl)-2-propyl-1-{[2'-(1H-1,2,3,4-tetrazol-5-yl)-[1,1'-biphenyl]-4-yl]methyl}-1H-imidazole-5-carboxylic acid | Azor, Benicar, Benicar Hct, Olmetec, Olmetec Plus, Tribenzor | C09CA08 | AGTR1 |
|  |  |  |  | azilsartan medoxomil | (5-methyl-2-oxo-2H-1,3-dioxol-4-yl)methyl 2-ethoxy-1-{[2'-(5-oxo-4,5-dihydro-1,2,4-oxadiazol-3-yl)-[1,1'-biphenyl]-4-yl]methyl}-1H-1,3-benzodiazole-7-carboxylate | Edarbi, Edarbyclor | C09CA09 | AGTR2 |
|  |  |  |  | fimasartan | 2-(2-butyl-4-methyl-6-oxo-1-{[2'-(1H-1,2,3,4-tetrazol-5-yl)-[1,1'-biphenyl]-4-yl]methyl}-1,6-dihydropyrimidin-5-yl)-N,N-dimethylethanethioamide | Kanarb | C09CA10 | AGTR3 |
|  |  | Renin-inhibitors | C09XA | remikiren | (2S)-2-[(2S)-2-benzyl-3-(2-methylpropane-2-sulfonyl)propanamido]-N-[(2S,3R,4S)-1-cyclohexyl-4-cyclopropyl-3,4-dihydroxybutan-2-yl]-3-(1H-imidazol-5-yl)propanamide |  | C09XA01 | REN |
|  |  |  |  | aliskiren | (2S,4S,5S,7S)-5-amino-N-(2-carbamoyl-2,2-dimethylethyl)-4-hydroxy-7-{[4-methoxy-3-(3-methoxypropoxy)phenyl]methyl}-8-methyl-2-(propan-2-yl)nonanamide | Rasilez, Tekturna, Tekturna Hct | C09XA02 | REN |
| CCBs | Selective calcium channel blockers with mainly vascular effects | Dihydropyridine derivatives | C08CA | amlodipine | 3-ethyl 5-methyl 2-[(2-aminoethoxy)methyl]-4-(2-chlorophenyl)-6-methyl-1,4-dihydropyridine-3,5-dicarboxylate | Amlobenz, Azor, Caduet, Dafiro, Exforge, Exforge Hct, Katerzia, Lotrel, Norliqva, Norvasc, Prestalia, Tribenzor, Twynsta, Viacoram | C08CA01 | CACNA1I, CA1, CACNB1, CACNA1C, SMPD1, CACNA1B, CACNA2D3 |
|  |  |  |  | felodipine | 3-ethyl 5-methyl 4-(2,3-dichlorophenyl)-2,6-dimethyl-1,4-dihydropyridine-3,5-dicarboxylate | Plendil | C08CA02 | CALM1, CACNA1C, NR3C2, CACNA2D1, CACNA1H, TNNC1, CACNB2, CACNA1S, CACNA1D, CACNA2D2, PDE1B, PDE1A, TNNC2 |
|  |  |  |  | isradipine | 3-methyl 5-propan-2-yl 4-(2,1,3-benzoxadiazol-4-yl)-2,6-dimethyl-1,4-dihydropyridine-3,5-dicarboxylate | DynaCirc, Prescal | C08CA03 | CACNA1C, CACNA2D1, CACNA1H, CACNB2, CACNA1S, CACNA1D, CACNA2D2 |
|  |  |  |  | nicardipine | 3-{2-[benzyl(methyl)amino]ethyl} 5-methyl 2,6-dimethyl-4-(3-nitrophenyl)-1,4-dihydropyridine-3,5-dicarboxylate | Cardene | C08CA04 | CHRM3, CHRM1, CHRM5, CHRM4, CALM1, CACNA1C, ADRA1A, CHRM2, ADRA1B, CACNA2D1, ADRA1D, CACNB2, CACNA1D, PDE1B, PDE1A |
|  |  |  |  | nifedipine | 3,5-dimethyl 2,6-dimethyl-4-(2-nitrophenyl)-1,4-dihydropyridine-3,5-dicarboxylate | Adalat, Afeditab CR, Nifediac, Nifedical, Procardia | C08CA05 | CALM1, CACNA1C, NR1I2, CACNB2, CACNA1S, CACNA1D, KCND3, CACNA1G, CACNA1H, CACNA1I |
|  |  |  |  | nimodipine | 3-(2-methoxyethyl) 5-propan-2-yl 2,6-dimethyl-4-(3-nitrophenyl)-1,4-dihydropyridine-3,5-dicarboxylate | Nimotop, Nymalize | C08CA06 | CACNB1, CACNA1C, NR3C2, CACNB2, CACNA1S, CACNB4, CACNA1F, CACNB3, CACNA1D, AHR |
|  |  |  |  | nisoldipine | 3-methyl 5-(2-methylpropyl) 2,6-dimethyl-4-(2-nitrophenyl)-1,4-dihydropyridine-3,5-dicarboxylate | Sular | C08CA07 | CACNA1C, CACNA2D1, CACNB2, CACNA1S, CACNA1D |
|  |  |  |  | nitrendipine | 3-ethyl 5-methyl 2,6-dimethyl-4-(3-nitrophenyl)-1,4-dihydropyridine-3,5-dicarboxylate | Baypress | C08CA08 | CACNA1C, CACNA2D1, CACNA1H, CACNB2, CACNA1S, CACNA1D, CACNA2D2 |
|  |  |  |  | lacidipine | 3,5-diethyl 4-{2-[(1E)-3-(tert-butoxy)-3-oxoprop-1-en-1-yl]phenyl}-2,6-dimethyl-1,4-dihydropyridine-3,5-dicarboxylate | Lacipil or Motens | C08CA09 | CACNA1C, CACNA1D, CACNA1F, CACNA1S, CACNB1, CACNB2, CACNB3, CACNB4, CACNA1A |
|  |  |  |  | nilvadipine | 3-methyl 5-propan-2-yl 2-cyano-6-methyl-4-(3-nitrophenyl)-1,4-dihydropyridine-3,5-dicarboxylate |  | C08CA10 | CACNA1C, CACNA2D1, CACNB2, CACNA1S, CACNA1D, CACNA2D3 |
|  |  |  |  | manidipine | 3-{2-[4-(diphenylmethyl)piperazin-1-yl]ethyl} 5-methyl 2,6-dimethyl-4-(3-nitrophenyl)-1,4-dihydropyridine-3,5-dicarboxylate | Manyper, Caldine | C08CA11 | CACNA1C, CACNA1D, CACNA1F, CACNA1S, CACNB1, CACNB2, CACNB3, CACNB4, CACNA1A, CACNA1G, CACNA1H, CACNA1I |
|  |  |  |  | barnidipine | 3-(3S)-1-benzylpyrrolidin-3-yl 5-methyl (4S)-2,6-dimethyl-4-(3-nitrophenyl)-1,4-dihydropyridine-3,5-dicarboxylate |  | C08CA12 |  |
|  |  |  |  | lercanidipine | 3-{1-[(3,3-diphenylpropyl)(methyl)amino]-2-methylpropan-2-yl} 5-methyl 2,6-dimethyl-4-(3-nitrophenyl)-1,4-dihydropyridine-3,5-dicarboxylate | Zanidip, Leridip | C08CA13 | CACNG1 |
|  |  |  |  | cilnidipine | 3-(2-methoxyethyl) 5-(2E)-3-phenylprop-2-en-1-yl 2,6-dimethyl-4-(3-nitrophenyl)-1,4-dihydropyridine-3,5-dicarboxylate | Atelec, Cilaheart, Cilacar | C08CA14 | CACNA1B, CACNA1C, CACNA1D, CACNA1F, CACNA1S, CACNB1, CACNB2, CACNB3, CACNB4, CACNA1A |
|  |  |  |  | benidipine | 3-(3R)-1-benzylpiperidin-3-yl 5-methyl (4R)-2,6-dimethyl-4-(3-nitrophenyl)-1,4-dihydropyridine-3,5-dicarboxylate |  | C08CA15 | CACNA1B, CACNA1C, CACNA1D, CACNA1F, CACNA1S, CACNB1, CACNB2, CACNB3, CACNB4, CACNA1A, CACNA1G, CACNA1H, CACNA1I |
|  |  |  |  | clevidipine | methyl 5-{[(butanoyloxy)methoxy]carbonyl}-4-(2,3-dichlorophenyl)-2,6-dimethyl-1,4-dihydropyridine-3-carboxylate | Cleviprex | C08CA16 | CACNA1C, CACNA1S, CACNA1F, CACNA1D |
|  |  | Other selective calcium channel blockers with mainly vascular effects | C08CX | mibefradil | (1S,2S)-2-(2-{[3-(1H-1,3-benzodiazol-2-yl)propyl](methyl)amino}ethyl)-6-fluoro-1-(propan-2-yl)-1,2,3,4-tetrahydronaphthalen-2-yl 2-methoxyacetate | Posicor | C08CX01 | CACNA1I, CACNB1, CACNA1C, CACNA1G, CACNA1H, CACNB2, CACNA1S, CACNB4, CACNA1F, CACNB3, CACNA1D |
|  | Selective calcium channel blockers with direct cardiac effects | Phenylalkylamine derivatives | C08DA | verapamil | 2-(3,4-dimethoxyphenyl)-5-{[2-(3,4-dimethoxyphenyl)ethyl](methyl)amino}-2-(propan-2-yl)pentanenitrile | Calan, Isoptin, Tarka, Verelan | C08DA01 | KCNH2, CACNA1A, CACNA1C, CACNA1G, ADRA1A, ADRA1B, KCNJ11, ADRA1D, SLC6A4, CACNA1H, CACNA1B |
|  |  |  |  | gallopamil | 5-{[2-(3,4-dimethoxyphenyl)ethyl](methyl)amino}-2-(propan-2-yl)-2-(3,4,5-trimethoxyphenyl)pentanenitrile |  | C08DA02 |  |
|  |  | Benzothiazepine derivatives | C08DB | diltiazem | (2S,3S)-5-[2-(dimethylamino)ethyl]-2-(4-methoxyphenyl)-4-oxo-2,3,4,5-tetrahydro-1,5-benzothiazepin-3-yl acetate | Cardizem, Cartia, Matzim, Taztia, Tiadylt, Tiazac | C08DB01 | CACNA1C, CACNG1 |
|  | Non-selective calcium channel blockers | Phenylalkylamine derivatives | C08EA | fendiline |  |  | C08EA01 |  |
|  |  |  |  | bepridil | N-benzyl-N-[3-(2-methylpropoxy)-2-(pyrrolidin-1-yl)propyl]aniline | Vascor | C08EA02 | KCNH2, CACNA1A, CALM1, KCNQ1, ATP1A1, CACNA1H, TNNC1, CACNA2D2, PDE1B, PDE1A |
|  |  | Other non-selective calcium channel blockers | C08EX | lidoflazine | 2-{4-[4,4-bis(4-fluorophenyl)butyl]piperazin-1-yl}-N-(2,6-dimethylphenyl)acetamide |  | C08EX01 |  |
|  |  |  |  | perhexiline | 2-(2,2-dicyclohexylethyl)piperidine |  | C08EX02 | KCNH2, CPT2, CPT1A |
| Beta blocker | Beta blocking agents | Beta blocking agents, non-selective | C07AA | alprenolol | 1-[2-(prop-2-en-1-yl)phenoxy]-3-[(propan-2-yl)amino]propan-2-ol | Gubernal, Regletin, Yobir, Apllobal, Aptine, Aptol Duriles | C07AA01 | ADRB1, HTR1A, ADRB2, ADRB3 |
|  |  |  |  | oxprenolol | 1-[2-(prop-2-en-1-yloxy)phenoxy]-3-[(propan-2-yl)amino]propan-2-ol | Oxprenolol | C07AA02 | ADRB1, ADRB2, ADRB3 |
|  |  |  |  | pindolol | 1-(1H-indol-4-yloxy)-3-[(propan-2-yl)amino]propan-2-ol | Viskazide, Visken | C07AA03 | ADRB1, HTR1A, ADRB2, HTR1B, ADRB3 |
|  |  |  |  | propranolol | 1-(naphthalen-1-yloxy)-3-[(propan-2-yl)amino]propan-2-ol | Hemangeol, Hemangiol, Inderal, Innopran | C07AA05 | ADRB1, HTR1A, ADRB2, HTR1B, ADRB3 |
|  |  |  |  | timolol | (2S)-1-(tert-butylamino)-3-{[4-(morpholin-4-yl)-1,2,5-thiadiazol-3-yl]oxy}propan-2-ol | Azarga, Betimol, Combigan, Cosopt, Duotrav, Istalol, Timoptic, Xalacom | C07AA06 | ADRB1, ADRB2, E |
|  |  |  |  | sotalol | N-(4-{1-hydroxy-2-[(propan-2-yl)amino]ethyl}phenyl)methanesulfonamide | Betapace, Sorine, Sotylize | C07AA07 | KCNH2, ADRB1, ADRB2 |
|  |  |  |  | nadolol | (2R,3S)-5-[3-(tert-butylamino)-2-hydroxypropoxy]-1,2,3,4-tetrahydronaphthalene-2,3-diol | Corgard | C07AA12 | ADRB1, ADRB2 |
|  |  |  |  | mepindolol | 1-[(2-methyl-1H-indol-4-yl)oxy]-3-[(propan-2-yl)amino]propan-2-ol | Visken | C07AA14 |  |
|  |  |  |  | carteolol | 5-[3-(tert-butylamino)-2-hydroxypropoxy]-1,2,3,4-tetrahydroquinolin-2-one | Ocupress | C07AA15 | ADRB1, ADRB2 |
|  |  |  |  | tertatolol | 1-(tert-butylamino)-3-(3,4-dihydro-2H-1-benzothiopyran-8-yloxy)propan-2-ol | Artex, Artexal, Prenalex | C07AA16 |  |
|  |  |  |  | bopindolol | 1-(tert-butylamino)-3-[(2-methyl-1H-indol-4-yl)oxy]propan-2-yl benzoate | pindolol | C07AA17 | ADRB1, HTR1A, ADRB2, HTR1B, ADRB3 |
|  |  |  |  | bupranolol | 1-(tert-butylamino)-3-(2-chloro-5-methylphenoxy)propan-2-ol |  | C07AA19 | ADRB1, ADRB2, ADRB3 |
|  |  |  |  | penbutolol | (2S)-1-(tert-butylamino)-3-(2-cyclopentylphenoxy)propan-2-ol | Levatol, Levatolol, Lobeta, Paginol, Hostabloc, Betapressin | C07AA23 | ADRB1, HTR1A, ADRB2, HTR1B |
|  |  |  |  | cloranolol | 1-(tert-butylamino)-3-(2,5-dichlorophenoxy)propan-2-ol |  | C07AA27 |  |
|  | Beta blocking agents | Beta blocking agents, selective | C07AB | practolol | N-(4-{2-hydroxy-3-[(propan-2-yl)amino]propoxy}phenyl)acetamide | Eraldin, Dalzic, Praktol, Cardiol, Pralon, Cordialina, Eraldina, Teranol | C07AB01 | ADRB1 |
|  |  |  |  | metoprolol | 1-[4-(2-methoxyethyl)phenoxy]-3-[(propan-2-yl)amino]propan-2-ol | Kapspargo, Lopressor, Lopressor Hct, Toprol | C07AB02 | ADRB1, ADRB2 |
|  |  |  |  | atenolol | 2-(4-{2-hydroxy-3-[(propan-2-yl)amino]propoxy}phenyl)acetamide | Tenoretic, Tenormin | C07AB03 | ADRB1, ADRB2 |
|  |  |  |  | acebutolol | N-(3-acetyl-4-{2-hydroxy-3-[(propan-2-yl)amino]propoxy}phenyl)butanamide | Sectral | C07AB04 | ADRB1, ADRB2 |
|  |  |  |  | betaxolol | 1-{4-[2-(cyclopropylmethoxy)ethyl]phenoxy}-3-[(propan-2-yl)amino]propan-2-ol | Betoptic, Betoptic Pilo, Betoptic S | C07AB05 | ADRB1, ADRB3 |
|  |  |  |  | bevantolol | 1-{[2-(3,4-dimethoxyphenyl)ethyl]amino}-3-(3-methylphenoxy)propan-2-ol |  | C07AB06 | ADRB1, ADRA1A, ADRB2 |
|  |  |  |  | bisoprolol | 1-[(propan-2-yl)amino]-3-(4-{[2-(propan-2-yloxy)ethoxy]methyl}phenoxy)propan-2-ol | Ziac | C07AB07 | ADRB1, ADRB2 |
|  |  |  |  | celiprolol | 1-{3-acetyl-4-[3-(tert-butylamino)-2-hydroxypropoxy]phenyl}-3,3-diethylurea |  | C07AB08 | ADRB1, ADRA2A, ADRA2C, ADRA2B, ADRB2, ADRB3 |
|  |  |  |  | esmolol | methyl 3-(4-{2-hydroxy-3-[(propan-2-yl)amino]propoxy}phenyl)propanoate | Brevibloc | C07AB09 | ADRB1 |
|  |  |  |  | epanolol | N-(2-{[3-(2-cyanophenoxy)-2-hydroxypropyl]amino}ethyl)-2-(4-hydroxyphenyl)acetamide | Visacor | C07AB10 |  |
|  |  |  |  | s-atenolol | 2-{4-[(2S)-2-hydroxy-3-[(propan-2-yl)amino]propoxy]phenyl}acetamide | Tenormin | C07AB11 |  |
|  |  |  |  | nebivolol | 1-(6-fluoro-3,4-dihydro-2H-1-benzopyran-2-yl)-2-{[2-(6-fluoro-3,4-dihydro-2H-1-benzopyran-2-yl)-2-hydroxyethyl]amino}ethan-1-ol | Nebilet, Bystolic | C07AB12 | ADRB1, ADRB2, ADRB3 |
|  |  |  |  | talinolol | 3-{4-[3-(tert-butylamino)-2-hydroxypropoxy]phenyl}-1-cyclohexylurea |  | C07AB13 |  |
|  |  |  |  | landiolol | [(4S)-2,2-dimethyl-1,3-dioxolan-4-yl]methyl 3-{4-[(2S)-2-hydroxy-3-({2-[(morpholine-4-carbonyl)amino]ethyl}amino)propoxy]phenyl}propanoate | Landiolol | C07AB14 |  |
|  | Beta blocking agents | Alpha and beta blocking agents | C07AG | labetalol | 2-hydroxy-5-{1-hydroxy-2-[(4-phenylbutan-2-yl)amino]ethyl}benzamide | Trandate | C07AG01 | ADRB1, ADRB2, ADRA1A, ADRA1B, ADRA1D |
|  |  |  |  | carvedilol | 1-(9H-carbazol-4-yloxy)-3-{[2-(2-methoxyphenoxy)ethyl]amino}propan-2-ol | Coreg | C07AG02 | KCNH2, VEGFA, ADRB1, NDUFC2, ADRA2A, ADRA2C, ADRA1A, ADRA2B, ADRA1B, ADRB2, ADRA1D, NPPB, SELE, GJA1, VCAM1, HIF1A, KCNJ4, KCNJ2 |
| Diuretic | Low-ceiling diuretics, thiazides | Thiazides, plain | C03AA | bendroflumethiazide | 3-benzyl-1,1-dioxo-6-(trifluoromethyl)-3,4-dihydro-2H-1lambda6,2,4-benzothiadiazine-7-sulfonamide | Bendroflumethiazide | C03AA01 | CA1, CA2, SLC12A3, CA4, KCNMA1 |
|  |  |  |  | hydroflumethiazide | 1,1-dioxo-6-(trifluoromethyl)-3,4-dihydro-2H-1lambda6,2,4-benzothiadiazine-7-sulfonamide | Saluron | C03AA02 | CA1, CA2, SLC12A1, CA4, KCNMA1, ATP1A1, CA12, CA9, CA7 |
|  |  |  |  | hydrochlorothiazide | 6-chloro-1,1-dioxo-3,4-dihydro-2H-1lambda6,2,4-benzothiadiazine-7-sulfonamide | Accuretic, Actelsar Hct, Aldactazide, Altace HCT, Atacand, Atacand Hct, Avalide, Benicar Hct, Diovan Hct, Exforge Hct, Hyzaar, Ifirmacombi, Karvezide, Lopressor Hct, Lotensin Hct, Maxzide, Micardis-hct, Olmetec Plus, Tekturna Hct, Teveten HCT, Tribenzor, Urozide, Vaseretic, Viskazide, Zestoretic, Ziac | C03AA03 | SLC12A3, KCNMA1 |
|  |  |  |  | chlorothiazide | 6-chloro-1,1-dioxo-4H-1lambda6,2,4-benzothiadiazine-7-sulfonamide | Diuril | C03AA04 | CA1, CA2, SLC12A3 |
|  |  |  |  | polythiazide | 6-chloro-2-methyl-1,1-dioxo-3-{[(2,2,2-trifluoroethyl)sulfanyl]methyl}-3,4-dihydro-2H-1lambda6,2,4-benzothiadiazine-7-sulfonamide | Minizide, Renese, Renese-R | C03AA05 | SLC12A3 |
|  |  |  |  | trichlormethiazide | 6-chloro-3-(dichloromethyl)-1,1-dioxo-3,4-dihydro-2H-1lambda6,2,4-benzothiadiazine-7-sulfonamide | Achletin, Diu-Hydrin, Triflumen | C03AA06 | CA1, CA2, SLC12A3, CA4, ATP1A1 |
|  |  |  |  | cyclopenthiazide | 6-chloro-3-(cyclopentylmethyl)-1,1-dioxo-3,4-dihydro-2H-1λ⁶,2,4-benzothiadiazine-7-sulfonamide | Navidrex | C03AA07 |  |
|  |  |  |  | methyclothiazide | 6-chloro-3-(chloromethyl)-2-methyl-1,1-dioxo-3,4-dihydro-2H-1lambda6,2,4-benzothiadiazine-7-sulfonamide | Aquatensen,  Enduron | C03AA08 | CA1, CA2, SLC12A1, CA4 |
|  |  |  |  | cyclothiazide | 3-{bicyclo[2.2.1]hept-5-en-2-yl}-6-chloro-1,1-dioxo-3,4-dihydro-2H-1lambda6,2,4-benzothiadiazine-7-sulfonamide | Anhydron, Acquirel, Doburil, Fluidil, Renazide, Tensodiural, Valmiran | C03AA09 | FXYD2, CA1, CA2, SFRP4, CA13, CA14, CA3, CA4, CA5A, CA5B, CA6, CA7, CA9 |
|  |  |  |  | mebutizide | 6-chloro-3-(3-methylpentan-2-yl)-1,1-dioxo-3,4-dihydro-2H-1lambda6,2,4-benzothiadiazine-7-sulfonamide | Neoniagar | C03AA13 |  |
|  | Low-ceiling diuretics, excl. thiazides | Sulfonamides, plain | C03BA | quinethazone | 7-chloro-2-ethyl-4-oxo-1,2,3,4-tetrahydroquinazoline-6-sulfonamide | Hydromox | C03BA02 | CA1, SLC12A2, CA2, SLC12A3, SLC12A1 |
|  |  |  |  | clopamide | 4-chloro-N-[(2R,6S)-2,6-dimethylpiperidin-1-yl]-3-sulfamoylbenzamide | clopamide | C03BA03 |  |
|  |  |  |  | chlortalidone | 2-chloro-5-(1-hydroxy-3-oxo-2,3-dihydro-1H-isoindol-1-yl)benzene-1-sulfonamide | Edarbyclor, Tenoretic, Thalitone | C03BA04 | CA1, SLC12A1 |
|  |  |  |  | mefruside | 4-chloro-N1-methyl-N1-[(2-methyloxolan-2-yl)methyl]benzene-1,3-disulfonamide | Baycaron | C03BA05 |  |
|  |  |  |  | clofenamide | 4-chlorobenzene-1,3-disulfonamide | Apetinil, ethylamphetamine | C03BA07 |  |
|  |  |  |  | metolazone | 7-chloro-2-methyl-3-(2-methylphenyl)-4-oxo-1,2,3,4-tetrahydroquinazoline-6-sulfonamide | Mykrox, Zaroxolyn | C03BA08 | SLC12A3 |
|  |  |  |  | meticrane | 6-methyl-1,1-dioxo-3,4-dihydro-2H-1λ⁶-benzothiopyran-7-sulfonamide |  | C03BA09 |  |
|  |  |  |  | xipamide | 4-chloro-N-(2,6-dimethylphenyl)-2-hydroxy-5-sulfamoylbenzamide | Aquaphor, Aquaphoril | C03BA10 |  |
|  |  |  |  | indapamide | 4-chloro-N-(2-methyl-2,3-dihydro-1H-indol-1-yl)-3-sulfamoylbenzamide | Natrilix, Indipam, Rawel, Tensaid, Alkapamid | C03BA11 | SLC12A3 |
|  |  |  |  | clorexolone | 6-chloro-2-cyclohexyl-3-oxo-2,3-dihydro-1H-isoindole-5-sulfonamide | Nefrolan | C03BA12 |  |
|  |  |  |  | fenquizone | 7-chloro-4-oxo-2-phenyl-1,2,3,4-tetrahydroquinazoline-6-sulfonamide |  | C03BA13 |  |
|  |  | Mercurial diuretics | C03BC | mersalyl | 2-(2-{[3-(hydroxymercurio)-2-methoxypropyl]carbamoyl}phenoxy)acetic acid | Acid, Mersalyl,  Mercuramide,  Mercusal,  Mersalin,  Mersalyl,  Mersalyl Acid,  Salyrgan. | C03BC01 | SLC16A1, AQP1, ALPL |
|  |  | Xanthine derivatives | C03BD | theobromine | 3,7-dimethyl-2,3,6,7-tetrahydro-1H-purine-2,6-dione |  | C03BD01 | ADORA1, PDE4B, ADORA2A |
|  |  | Other low-ceiling diuretics | C03BX | cicletanine | 3-(4-chlorophenyl)-6-methyl-1H,3H-furo[3,4-c]pyridin-7-ol | Tenstaten | C03BX03 |  |
|  | High-ceiling diuretics | Sulfonamides, plain | C03CA | furosemide | 4-chloro-2-{[(furan-2-yl)methyl]amino}-5-sulfamoylbenzoic acid | Furoscix, Lasix | C03CA01 | CA2, SLC12A1, GPR35 |
|  |  |  |  | bumetanide | 3-(butylamino)-4-phenoxy-5-sulfamoylbenzoic acid | Bumex, Burinex | C03CA02 | SLC12A5, SLC12A2, SLC12A1, SLC12A4, CFTR |
|  |  |  |  | piretanide | 4-phenoxy-3-(pyrrolidin-1-yl)-5-sulfamoylbenzoic acid | Arelix, Eurelix, Tauliz | C03CA03 | SLC12A1 |
|  |  |  |  | torasemide | 1-({4-[(3-methylphenyl)amino]pyridin-3-yl}sulfonyl)-3-(propan-2-yl)urea | Demadex, Soaanz | C03CA04 | SLC12A2, SLC12A1 |
|  |  | Aryloxyacetic acid derivatives | C03CC | etacrynic acid | 2-[2,3-dichloro-4-(2-methylidenebutanoyl)phenoxy]acetic acid |  | C03CC01 | SLC12A1, ATP1A1, GSTP1, LEF1 |
|  |  |  |  | tienilic acid | 2-[2,3-dichloro-4-(thiophene-2-carbonyl)phenoxy]acetic acid |  | C03CC02 |  |
|  |  | Pyrazolone derivatives | C03CD | muzolimine | 3-amino-1-[1-(3,4-dichlorophenyl)ethyl]-4,5-dihydro-1H-pyrazol-5-one |  | C03CD01 |  |
|  |  | Other high-ceiling diuretics | C03CX | etozolin |  |  | C03CX01 |  |
|  | Potassium-sparing agents | Aldosterone antagonists | C03DA | spironolactone | (1R,3aS,3bR,4R,9aR,9bS,11aS)-4-(acetylsulfanyl)-9a,11a-dimethyl-2,3,3a,3b,4,5,7,8,9,9a,9b,10,11,11a-tetradecahydrospiro[cyclopenta[a]phenanthrene-1,2'-oxolane]-5',7-dione | Aldactazide, Aldactone, Carospir | C03DA01 | AR, CACNA1A, CACNA1B, CACNA1C, CACNA1D, CACNA1E, CACNA1F, CACNA1G, CACNA1H, CACNA1I, CACNA1S, CACNA2D1, CACNA2D2, CACNA2D3, CACNA2D4, CACNB1, CACNB2, CACNB3, CACNB4, CACNG1, CACNG2, CACNG3, CACNG4, CACNG5, CACNG6, CACNG7, CACNG8, CYP11B2, CYP17A1, NR1I2, NR3C1, NR3C2, PGR, SHBG, SRD5A1, SRD5A2, SRD5A3 |
|  |  |  |  | potassium canrenoate | 3-[(1S,2R,10R,11S,14R,15S)-14-hydroxy-2,15-dimethyl-5-oxotetracyclo[8.7.0.0²,⁷.0¹¹,¹⁵]heptadeca-6,8-dien-14-yl]propanoic acid | Venactone, Soldactone | C03DA02 |  |
|  |  |  |  | canrenone | (1R,3aS,3bR,9aR,9bS,11aS)-9a,11a-dimethyl-2,3,3a,3b,7,8,9,9a,9b,10,11,11a-dodecahydrospiro[cyclopenta[a]phenanthrene-1,2'-oxolane]-5',7-dione | Contaren, Luvion, Phanurane, Spiroletan | C03DA03 |  |
|  |  |  |  | eplerenone | methyl (1'R,2R,2'S,9'R,10'R,11'S,15'S,17'R)-2',15'-dimethyl-5,5'-dioxo-18'-oxaspiro[oxolane-2,14'-pentacyclo[8.8.0.0^{1,17}.0^{2,7}.0^{11,15}]octadecan]-6'-ene-9'-carboxylate | Inspra | C03DA04 | NR3C2 |
|  |  | Other potassium-sparing agents | C03DB | amiloride | 3,5-diamino-N-carbamimidoyl-6-chloropyrazine-2-carboxamide | Midamor | C03DB01 | AOC1, ASIC1, ASIC2, PLAU, SCNN1A, SCNN1B, SCNN1D, SCNN1G, SLC9A1 |
|  |  |  |  | triamterene | 6-phenylpteridine-2,4,7-triamine | Dyrenium, Maxzide | C03DB02 | SCNN1A, SCNN1B, SCNN1D, SCNN1G |
|  | Other diuretics | Vasopressin antagonists | C03XA | tolvaptan | N-{4-[(5R)-7-chloro-5-hydroxy-2,3,4,5-tetrahydro-1H-1-benzazepine-1-carbonyl]-3-methylphenyl}-2-methylbenzamide | Jinarc, Jynarque 45/15 Carton, Samsca | C03XA01 | AVPR1A, AVPR2 |
|  |  |  |  | conivaptan | N-(4-{4-methyl-3,5,9-triazatricyclo[8.4.0.0^{2,6}]tetradeca-1(14),2(6),3,10,12-pentaene-9-carbonyl}phenyl)-[1,1'-biphenyl]-2-carboxamide | Vaprisol | C03XA02 | AVPR1A, AVPR3 |

IUPAC, the IUPAC nomenclature of organic chemistry is a method of naming organic chemical compounds as recommended by the International Union of Pure and Applied Chemistry (IUPAC).

**Supplementary File 1c** MR association between drug target gene expression in blood and systolic blood pressure

| **Gene** | **ProbeChr** | **Probe_bp** | **topSNP** | **topSNP_chr** | **topSNP_bp** | **Effect_allele** | **Other_allele** | **Freq_Effect_allele** | **eQTL association** | | | | **SBP association** | | | **MR association** | | | **HEIDI Test** | |
| --- | --- | --- | --- | --- | --- | --- | --- | --- | --- | --- | --- | --- | --- | --- | --- | --- | --- | --- | --- | --- |
|  |  |  |  |  |  |  |  |  | **beta** | **se** | **p** | **F_statistics** | **beta** | **se** | **p** | **beta** | **se** | **p** | **p_HEIDI** | **nsnp** |
| P4HA1 | 10 | 74811853 | rs6480668 | 10 | 74849326 | G | A | 0.07 | 0.183 | 0.016 | 3.35E-32 | 139.5 | 0.304 | 0.061 | 7.17E-07 | 1.655 | 0.362 | 4.82E-06 | 4.41E-02 | 3 |
| ACE | 17 | 61576813 | rs4308 | 17 | 61559625 | A | G | 0.38 | 0.075 | 0.009 | 2.84E-17 | 71.5 | 0.273 | 0.031 | 3.11E-18 | 3.639 | 0.600 | 1.35E-09 | 1.10E-01 | 17 |
| SLC12A2 | 5 | 127472419 | rs17764730 | 5 | 127357526 | T | C | 0.22 | -0.186 | 0.009 | 3.2E-86 | 387.3 | -0.208 | 0.035 | 4.49E-09 | 1.119 | 0.199 | 1.85E-08 | 4.27E-02 | 20 |
| AOC1 | 7 | 150540153 | rs7806458 | 7 | 150476888 | G | A | 0.36 | 0.669 | 0.008 | 0.00E+00 | 6991.5 | 0.078 | 0.031 | 1.27E-02 | 0.117 | 0.047 | 1.28E-02 | 9.06E-02 | 20 |
| SCNN1D | 1 | 1221612 | rs11804831 | 1 | 1194804 | C | T | 0.15 | -0.133 | 0.012 | 4.69E-30 | 129.7 | -0.161 | 0.041 | 6.90E-05 | 1.208 | 0.321 | 1.72E-04 | 1.13E-02 | 20 |
| SLC12A1 | 15 | 48540068 | rs964611 | 15 | 48597514 | A | C | 0.15 | 1.664 | 0.009 | 0.00E+00 | 37527.1 | -0.093 | 0.042 | 2.52E-02 | -0.056 | 0.025 | 2.53E-02 | 4.70E-01 | 20 |
| CA4 | 17 | 58237778 | rs34820870 | 17 | 58244021 | G | T | 0.03 | -0.462 | 0.021 | 6.1E-110 | 496.3 | 0.202 | 0.085 | 1.74E-02 | -0.438 | 0.185 | 1.80E-02 | 1.64E-02 | 20 |
| SLC16A1 | 1 | 113477052 | rs3789592 | 1 | 113471400 | A | G | 0.41 | 0.123 | 0.008 | 4.34E-53 | 235.2 | -0.178 | 0.031 | 6.28E-09 | -1.443 | 0.266 | 5.61E-08 | 6.64E-01 | 20 |
| ADRB2 | 5 | 148207176 | rs2082395 | 5 | 148200600 | A | G | 0.42 | 0.126 | 0.008 | 6.31E-56 | 248.2 | -0.119 | 0.030 | 8.83E-05 | -0.944 | 0.248 | 1.42E-04 | 7.66E-01 | 5 |
| KCNJ11 | 11 | 17409142 | rs2074310 | 11 | 17421886 | T | C | 0.37 | 0.157 | 0.012 | 4.37E-38 | 166.5 | 0.340 | 0.032 | 5.50E-27 | 2.160 | 0.262 | 1.51E-16 | 6.53E-02 | 20 |
| ATP1A1 | 1 | 116934086 | rs6704439 | 1 | 116867616 | A | C | 0.27 | 0.041 | 0.009 | 4.39E-06 | 21.1 | 0.147 | 0.035 | 2.39E-05 | 3.567 | 1.148 | 1.88E-03 | 2.09E-01 | 8 |
| SLC9A1 | 1 | 27459389 | rs12751422 | 1 | 27493679 | T | C | 0.27 | 0.106 | 0.009 | 1.19E-32 | 141.6 | -0.148 | 0.035 | 2.08E-05 | -1.395 | 0.348 | 6.08E-05 | 9.72E-02 | 20 |
| NR3C1 | 5 | 142736286 | rs4912908 | 5 | 142791133 | G | A | 0.23 | 0.073 | 0.010 | 1.07E-13 | 55.2 | 0.094 | 0.038 | 1.20E-02 | 1.296 | 0.545 | 1.74E-02 | 2.92E-01 | 15 |
| CACNA1H | 16 | 1237506 | rs35198836 | 16 | 1244631 | T | C | 0.22 | 0.149 | 0.011 | 2.95E-39 | 171.8 | 0.099 | 0.035 | 4.71E-03 | 0.668 | 0.241 | 5.69E-03 | 3.96E-01 | 20 |
| AQP1 | 7 | 30929070 | rs28362721 | 7 | 30957702 | T | C | 0.17 | 0.086 | 0.011 | 1.81E-15 | 63.3 | 0.115 | 0.041 | 4.93E-03 | 1.345 | 0.507 | 7.97E-03 | 5.12E-01 | 20 |
| JUN | 1 | 59248125 | rs2716140 | 1 | 59472397 | C | A | 0.40 | -0.228 | 0.008 | 3.5E-177 | 805.5 | 0.126 | 0.031 | 4.63E-05 | -0.551 | 0.137 | 5.58E-05 | 9.09E-02 | 20 |
| CA12 | 15 | 63643968 | rs12909041 | 15 | 63743677 | A | C | 0.20 | 0.039 | 0.010 | 0.000114 | 14.9 | 0.090 | 0.038 | 1.92E-02 | 2.319 | 1.158 | 4.53E-02 | 1.08E-02 | 5 |
| CACNA1D | 3 | 53687586 | rs9830632 | 3 | 53735766 | G | A | 0.29 | -0.085 | 0.009 | 2E-22 | 94.9 | 0.207 | 0.034 | 9.24E-10 | -2.427 | 0.468 | 2.14E-07 | 3.93E-01 | 20 |
| CACNA2D2 | 3 | 50470954 | rs62260815 | 3 | 50474624 | A | G | 0.11 | -0.142 | 0.013 | 8.98E-29 | 123.9 | -0.116 | 0.049 | 1.67E-02 | 0.814 | 0.348 | 1.94E-02 | 7.93E-02 | 20 |
| PDE1B | 12 | 54958078 | rs1874308 | 12 | 54954356 | A | T | 0.30 | 0.429 | 0.009 | 0.00E+00 | 2301.7 | -0.094 | 0.034 | 5.33E-03 | -0.220 | 0.079 | 5.40E-03 | 7.35E-01 | 20 |
| AHR | 7 | 17362011 | rs17700436 | 7 | 17167825 | T | C | 0.05 | 0.480 | 0.017 | 1.6E-178 | 811.7 | -0.184 | 0.066 | 5.34E-03 | -0.383 | 0.138 | 5.58E-03 | 7.08E-01 | 20 |
| GPR35 | 2 | 241557762 | rs2975788 | 2 | 241571858 | G | A | 0.48 | 0.293 | 0.009 | 6.2E-259 | 1181.6 | 0.065 | 0.030 | 3.29E-02 | 0.221 | 0.104 | 3.34E-02 | 7.59E-01 | 20 |
| KCNJ2 | 17 | 68170501 | rs9890133 | 17 | 68169005 | G | A | 0.11 | -0.256 | 0.012 | 8.02E-94 | 422.2 | 0.094 | 0.047 | 4.61E-02 | -0.367 | 0.185 | 4.72E-02 | 6.24E-01 | 20 |

Beta of the eQTL association is the standard deviation change in gene expression per coded allele. Beta of the BC association is the log odds per coded allele. Beta of MR association represents the mmHg change of blood pressure per 1SD increase in expression.

Abbreviations: MR, Mendelian randomization, SNP, single nuclear polymorphism, eQTL, expression quantitative trait loci, se, standard error, SBP, systolic blood pressure, HEIDI, heterogeneity in dependent instruments, nsnp, number of SNPs for HEIDI test.

**Supplementary File 1d** Association between SLC12A2 and PDE1B eQTL SNP in blood with expression of other nearby genes

|  | **Nearby gene** | **Chr** | **BP** | **Samples** | **eQTL SNP** | **SNP_BP** | **Effect_allele** | **Other_allele** | **Freq_Effect_allele** | **beta** | **se** | **p** |
| --- | --- | --- | --- | --- | --- | --- | --- | --- | --- | --- | --- | --- |
| SLC12A2 | **CTC-228N24.3** | **5** | **127347455** | **22495** | **rs17764730** | **127357526** | **T** | **C** | **0.227** | **-0.503** | **0.011** | **0.00E+00** |
|  | **SLC12A2** | **5** | **127472419** | **31644** | **rs17764730** | **127357526** | **T** | **C** | **0.227** | **-0.186** | **0.009** | **3.20E-86** |
|  | **FBN2** | **5** | **127794239** | **31684** | **rs17764730** | **127357526** | **T** | **C** | **0.227** | **-0.158** | **0.009** | **6.90E-63** |
|  | PRRC1 | 5 | 126872041 | 31684 | rs17764730 | 127357526 | T | C | 0.227 | -0.019 | 0.009 | 5.01E-02 |
|  | CTC-228N24.1 | 5 | 127158204 | 15590 | rs17764730 | 127357526 | T | C | 0.227 | 0.013 | 0.014 | 3.37E-01 |
|  | HNRNPKP1 | 5 | 126847845 | 19056 | rs17764730 | 127357526 | T | C | 0.227 | -0.004 | 0.012 | 7.30E-01 |
|  | C5orf63 | 5 | 126393717 | 25262 | rs17764730 | 127357526 | T | C | 0.227 | 0.003 | 0.011 | 7.81E-01 |
| PDE1B | **PDE1B** | **12** | **54958078** | **28131** | **rs1874308** | **54954356** | **A** | **T** | **0.289** | **0.429** | **0.009** | **0.00E+00** |
|  | **NCKAP1L** | **12** | **54914610** | **28131** | **rs1874308** | **54954356** | **A** | **T** | **0.289** | **0.038** | **0.009** | **5.00E-05** |
|  | **RP11-834C11.3** | **12** | **54484028** | **3827** | **rs1874308** | **54954356** | **A** | **T** | **0.289** | **-0.077** | **0.025** | **2.15E-03** |
|  | **GPR84** | **12** | **54757250** | **28131** | **rs1874308** | **54954356** | **A** | **T** | **0.289** | **-0.026** | **0.009** | **4.92E-03** |
|  | **SMUG1** | **12** | **54570653** | **27382** | **rs1874308** | **54954356** | **A** | **T** | **0.289** | **0.022** | **0.009** | **1.83E-02** |
|  | RP11-1049A21.2 | 12 | 54937392 | 4652 | rs1874308 | 54954356 | A | T | 0.289 | -0.045 | 0.023 | 5.07E-02 |
|  | RP11-968A15.8 | 12 | 54704641 | 3827 | rs1874308 | 54954356 | A | T | 0.289 | -0.047 | 0.025 | 6.18E-02 |
|  | HNRNPA1 | 12 | 54677424 | 23056 | rs1874308 | 54954356 | A | T | 0.289 | -0.014 | 0.010 | 1.66E-01 |
|  | NFE2 | 12 | 54690400 | 28131 | rs1874308 | 54954356 | A | T | 0.289 | 0.013 | 0.009 | 1.79E-01 |
|  | RP11-968A15.2 | 12 | 54664623 | 4652 | rs1874308 | 54954356 | A | T | 0.289 | 0.031 | 0.023 | 1.82E-01 |
|  | ATF7 | 12 | 53960919 | 11154 | rs1874308 | 54954356 | A | T | 0.289 | 0.019 | 0.015 | 1.97E-01 |
|  | RP11-753H16.5 | 12 | 54804172 | 4018 | rs1874308 | 54954356 | A | T | 0.289 | 0.029 | 0.025 | 2.34E-01 |
|  | ITGA5 | 12 | 54801144 | 28131 | rs1874308 | 54954356 | A | T | 0.289 | 0.011 | 0.009 | 2.52E-01 |
|  | ZNF385A | 12 | 54773999 | 28131 | rs1874308 | 54954356 | A | T | 0.289 | -0.010 | 0.009 | 2.67E-01 |
|  | RP11-834C11.4 | 12 | 54523254 | 21824 | rs1874308 | 54954356 | A | T | 0.289 | -0.011 | 0.011 | 2.86E-01 |
|  | PHC1P1 | 12 | 55806448 | 4526 | rs1874308 | 54954356 | A | T | 0.289 | -0.024 | 0.023 | 3.03E-01 |
|  | ATP5G2 | 12 | 54048851 | 28131 | rs1874308 | 54954356 | A | T | 0.289 | -0.009 | 0.009 | 3.60E-01 |
|  | HOXC5 | 12 | 54404387 | 27306 | rs1874308 | 54954356 | A | T | 0.289 | -0.008 | 0.009 | 3.91E-01 |
|  | COPZ1 | 12 | 54720309 | 28131 | rs1874308 | 54954356 | A | T | 0.289 | -0.006 | 0.009 | 5.00E-01 |
|  | HOXC4 | 12 | 54430264 | 22231 | rs1874308 | 54954356 | A | T | 0.289 | 0.007 | 0.010 | 5.21E-01 |
|  | RP11-834C11.7 | 12 | 54473154 | 10394 | rs1874308 | 54954356 | A | T | 0.289 | -0.009 | 0.015 | 5.55E-01 |
|  | CBX5 | 12 | 54649305 | 27917 | rs1874308 | 54954356 | A | T | 0.289 | -0.004 | 0.009 | 6.70E-01 |
|  | TESPA1 | 12 | 55360166 | 21824 | rs1874308 | 54954356 | A | T | 0.289 | -0.004 | 0.011 | 6.97E-01 |
|  | GTSF1 | 12 | 54858560 | 11154 | rs1874308 | 54954356 | A | T | 0.289 | 0.006 | 0.015 | 7.09E-01 |
|  | CALCOCO1 | 12 | 54113216 | 28131 | rs1874308 | 54954356 | A | T | 0.289 | -0.003 | 0.009 | 7.19E-01 |

Abbreviations: SNP, single nuclear polymorphism, eQTL, expression quantitative trait loci, se, standard error.

**Supplementary File 1e** MR association between expression of nearby genes of SLC12A2 and PDE1B in blood and risk of breast cancer and ER+ breast cancer

| **BC type** | **Target gene** | **Nearby gene** | **ProbeChr** | **Probe bp** | **topSNP** | **topSNP_bp** | **Effect allele** | **Other allele** | **Freq Effect allele** | **eQTL association** | | | **BC association** | | | **MR association** | | | **HEIDI Test** | | **Posterior probability** |
| --- | --- | --- | --- | --- | --- | --- | --- | --- | --- | --- | --- | --- | --- | --- | --- | --- | --- | --- | --- | --- | --- |
|  |  |  |  |  |  |  |  |  |  | **beta** | **se** | **p** | **beta** | **se** | **p** | **beta** | **se** | **p** | **p_HEIDI** | **nsnp** |  |
| Any BC | SLC12A2 | CTC-228N24.3 | 5 | 127347455 | rs6888037 | 127406259 | G | T | 0.21 | -0.508 | 0.011 | 0.00E+00 | 0.027 | 0.007 | 1.09E-04 | -0.054 | 0.014 | 1.15E-04 | 1.45E-01 | 20 | 77% |
|  |  | SLC12A2 | 5 | 127472419 | rs17764730 | 127357526 | T | C | 0.21 | -0.186 | 0.009 | 3.20E-86 | 0.028 | 0.007 | 7.57E-05 | -0.150 | 0.039 | 1.05E-04 | 1.56E-01 | 20 | 81.50% |
|  |  | FBN2 | 5 | 127794239 | rs79813368 | 127921198 | A | G | 0.05 | 1.262 | 0.013 | 0.00E+00 | -0.003 | 0.011 | 7.71E-01 | -0.002 | 0.009 | 7.71E-01 | 9.98E-01 | 20 | 0.33% |
| ER+ BC | SLC12A2 | CTC-228N24.3 | 5 | 127347455 | rs6888037 | 127406259 | G | T | 0.21 | -0.508 | 0.011 | 0.00E+00 | 0.029 | 0.009 | 7.99E-04 | -0.057 | 0.017 | 8.48E-04 | 1.70E-01 | 20 | 40.40% |
|  |  | SLC12A2 | 5 | 127472419 | rs17764730 | 127357526 | T | C | 0.21 | -0.186 | 0.009 | 3.20E-86 | 0.029 | 0.009 | 6.96E-04 | -0.158 | 0.048 | 8.67E-04 | 1.23E-01 | 20 | 40.46% |
|  |  | FBN2 | 5 | 127794239 | rs79813368 | 127921198 | A | G | 0.05 | 1.262 | 0.013 | 0.00E+00 | -0.004 | 0.013 | 7.78E-01 | -0.003 | 0.011 | 7.75E-01 | 1.00E+00 | 20 | 0.42% |
|  | PDE1B | PDE1B | 12 | 54958078 | rs1874308 | 54954356 | A | T | 0.39 | 0.429 | 0.009 | 0.00E+00 | 0.030 | 0.008 | 3.38E-04 | 0.070 | 0.019 | 3.28E-04 | 2.37E-01 | 14 | 66.80% |
|  |  | NCKAP1L | 12 | 54914610 | rs11609712 | 54890515 | A | C | 0.12 | 0.187 | 0.009 | 5.93E-100 | 0.010 | 0.009 | 2.69E-01 | 0.053 | 0.049 | 2.72E-01 | 1.03E-01 | 20 | 0.62% |
|  |  | GPR84 | 12 | 54757250 | rs3809161 | 54758330 | A | G | 0.50 | 0.222 | 0.008 | 3.93E-175 | -0.015 | 0.008 | 6.21E-02 | -0.067 | 0.036 | 6.16E-02 | 4.08E-01 | 20 | 1.67% |
|  |  | SMUG1 | 12 | 54570653 | rs2233921 | 54575800 | A | C | 0.32 | -0.344 | 0.008 | 0.00E+00 | -0.005 | 0.008 | 5.29E-01 | 0.014 | 0.022 | 5.31E-01 | 1.42E-01 | 20 | 0.34% |

Beta of the eQTL association is the standard deviation change in gene expression per coded allele. Beta of the BC association is the log odds per coded allele. Beta of MR association represents the log odds per one standard deviation increase in gene expression. A significant HEIDI p-value (<0.01) indicates that any association between gene expression and outcome may be due to linkage where there are two distinct causal variants in linkage disequilibrium.

Abbreviations: MR, Mendelian randomization, SNP, single nuclear polymorphism, eQTL, expression quantitative trait loci, se, standard error, BC, breast cancer, HEIDI, heterogeneity in dependent instruments, nsnp, number of SNPs for HEIDI test.

**Supplementary File 1f** MR association between drug targeted gene expression in other tissue and overall risk of breast cancer

| **Tissue** | **Gene** | **Gene_chr** | **Gene_start** | **topSNP** | **topSNP_chr** | **topSNP_bp** | **Effect_allele** | **Other_allele** | **Freq_Effect_allele** | **eQTL association** | | | **BC association** | | | **MR association** | | | **HEIDI Test** | |
| --- | --- | --- | --- | --- | --- | --- | --- | --- | --- | --- | --- | --- | --- | --- | --- | --- | --- | --- | --- | --- |
|  |  |  |  |  |  |  |  |  |  | **beta** | **se** | **p** | **beta** | **se** | **p** | **beta** | **se** | **p** | **p_HEIDI** | **nsnp** |
| Adrenal_Gland | PDE1B | 12 | 54943134 | rs3782410 | 12 | 54967739 | A | G | 0.337 | -0.240 | 0.067 | 3.37E-04 | 0.020 | 0.007 | 2.81E-03 | -0.083 | 0.036 | 2.17E-02 | NA | NA |
|  | CACNA1D | 3 | 53528683 | rs41292864 | 3 | 52852410 | A | G | 0.041 | -0.371 | 0.083 | 7.98E-06 | -0.022 | 0.010 | 3.60E-02 | 0.059 | 0.031 | 5.77E-02 | 9.18E-01 | 19 |
|  | SLC12A2 | 5 | 127419458 | rs247210 | 5 | 128086098 | A | G | 0.370 | -0.133 | 0.047 | 4.47E-03 | -0.014 | 0.006 | 2.15E-02 | 0.106 | 0.059 | 7.39E-02 | NA | NA |
|  | SLC12A1 | 15 | 48483861 | rs148775372 | 15 | 49202279 | AAGGTATGCT | A | 0.081 | -0.977 | 0.266 | 2.35E-04 | 0.042 | 0.021 | 4.26E-02 | -0.043 | 0.024 | 7.58E-02 | 5.18E-01 | 5 |
| Breast_Mammary | PDE1B | 12 | 54943134 | rs10747699 | 12 | 54955324 | C | T | 0.606 | 0.310 | 0.027 | 9.16E-30 | -0.017 | 0.007 | 1.31E-02 | -0.054 | 0.022 | 1.53E-02 | NA | NA |
|  | CACNA2D2 | 3 | 50400233 | rs62260389 | 3 | 51163442 | G | T | 0.053 | -0.191 | 0.057 | 7.56E-04 | -0.023 | 0.009 | 8.28E-03 | 0.119 | 0.057 | 3.77E-02 | NA | NA |
|  | ADRB2 | 5 | 148206156 | rs4077085 | 5 | 148544045 | G | A | 0.380 | -0.072 | 0.023 | 2.08E-03 | -0.016 | 0.006 | 6.26E-03 | 0.227 | 0.111 | 4.09E-02 | NA | NA |
|  | KCNJ2 | 17 | 68164814 | rs2189592 | 17 | 68005334 | C | T | 0.978 | -0.240 | 0.069 | 5.05E-04 | 0.027 | 0.014 | 4.95E-02 | -0.111 | 0.065 | 8.72E-02 | 9.96E-01 | 4 |
|  | SCNN1D | 1 | 1215816 | rs11260570 | 1 | 1201640 | C | T | 0.099 | 0.392 | 0.058 | 2.12E-11 | -0.030 | 0.018 | 8.72E-02 | -0.077 | 0.046 | 9.75E-02 | 9.25E-01 | 20 |
| Kidney_Cortex | KCNJ2 | 17 | 68164814 | rs8081686 | 17 | 67795333 | C | T | 0.16 | -0.795 | 0.222 | 3.35E-04 | -0.038 | 0.011 | 4.51E-04 | 0.048 | 0.019 | 1.22E-02 | NA | NA |
|  | NR3C1 | 5 | 142657496 | rs71587225 | 5 | 141999683 | T | C | 0.18 | 0.443 | 0.109 | 5.03E-05 | 0.025 | 0.008 | 1.65E-03 | 0.056 | 0.023 | 1.29E-02 | 5.47E-01 | 6 |
|  | SLC16A1 | 1 | 113454469 | rs1343273 | 1 | 112875024 | A | G | 0.57 | -0.393 | 0.106 | 2.09E-04 | 0.020 | 0.007 | 4.42E-03 | -0.051 | 0.023 | 2.39E-02 | NA | NA |
|  | AOC1 | 7 | 150521715 | rs111645229 | 7 | 150567565 | A | C | 0.02 | -1.583 | 0.350 | 6.08E-06 | 0.039 | 0.016 | 1.54E-02 | -0.025 | 0.012 | 3.27E-02 | 4.83E-01 | 20 |
|  | ADRB2 | 5 | 148206156 | rs56296220 | 5 | 148116014 | A | G | 0.05 | -1.005 | 0.306 | 1.02E-03 | 0.058 | 0.027 | 3.09E-02 | -0.058 | 0.032 | 7.13E-02 | 5.77E-01 | 5 |
|  | PDE1B | 12 | 54943134 | rs10876556 | 12 | 54758960 | A | G | 0.36 | -0.350 | 0.135 | 9.71E-03 | 0.015 | 0.006 | 1.48E-02 | -0.044 | 0.025 | 7.62E-02 | NA | NA |
|  | CACNA1H | 16 | 1203241 | rs72761142 | 16 | 1663250 | A | T | 0.06 | -0.700 | 0.200 | 4.64E-04 | 0.021 | 0.011 | 4.63E-02 | -0.031 | 0.018 | 8.33E-02 | NA | NA |
| Muscle_Skeletal | JUN | 1 | 59246465 | rs706419 | 1 | 58887296 | A | G | 0.538 | 0.152 | 0.037 | 4.47E-05 | -0.013 | 0.006 | 3.25E-02 | -0.087 | 0.046 | 5.83E-02 | 4.95E-01 | 13 |
|  | CACNA2D2 | 3 | 50400233 | rs2236971 | 3 | 50473349 | A | G | 0.179 | -0.125 | 0.043 | 3.95E-03 | 0.019 | 0.009 | 3.00E-02 | -0.154 | 0.089 | 8.30E-02 | NA | NA |
|  | SCNN1D | 1 | 1215816 | rs113867217 | 1 | 1214805 | T | C | 0.101 | 0.278 | 0.042 | 3.73E-11 | -0.030 | 0.018 | 8.62E-02 | -0.109 | 0.066 | 9.68E-02 | 9.75E-01 | 20 |
|  | P4HA1 | 10 | 74766975 | rs58907430 | 10 | 74745364 | C | A | 0.183 | 0.168 | 0.045 | 1.68E-04 | -0.021 | 0.012 | 7.79E-02 | -0.127 | 0.080 | 1.10E-01 | 2.00E-01 | 3 |
| Small_Intestine_Terminal_Ileum | PDE1B | 12 | 54943134 | rs17109031 | 12 | 54598367 | A | G | 0.20 | 0.165 | 0.059 | 5.57E-03 | -0.022 | 0.010 | 2.80E-02 | -0.133 | 0.077 | 8.51E-02 | NA | NA |
|  | ADRB2 | 5 | 148206156 | rs11740127 | 5 | 148305690 | C | T | 0.21 | 0.191 | 0.054 | 3.75E-04 | 0.013 | 0.007 | 5.27E-02 | 0.067 | 0.039 | 8.89E-02 | NA | NA |
| Uterus | ADRB2 | 5 | 148206156 | rs36079 | 5 | 148430504 | A | G | 0.33 | 0.401 | 0.114 | 4.27E-04 | 0.031 | 0.007 | 2.49E-05 | 0.078 | 0.029 | 6.87E-03 | NA | NA |
|  | KCNJ11 | 11 | 17407406 | rs873314 | 11 | 17462858 | C | T | 0.18 | -0.482 | 0.113 | 2.01E-05 | -0.020 | 0.008 | 9.30E-03 | 0.041 | 0.018 | 2.64E-02 | NA | NA |
|  | CACNA1H | 16 | 1203241 | rs2072950 | 16 | 1510567 | G | T | 0.67 | 0.308 | 0.084 | 2.36E-04 | -0.015 | 0.007 | 2.57E-02 | -0.047 | 0.025 | 5.64E-02 | NA | NA |

Beta of the eQTL association is the standard deviation change in gene expression per coded allele. Beta of the BC association is the log odds per coded allele. Beta of MR association represents the log odds per one standard deviation increase in gene expression. A significant HEIDI p-value (<0.01) indicates that any association between gene expression and outcome may be due to linkage where there are two distinct causal variants in linkage disequilibrium.

MR, Mendelian randomization, SNP, single nuclear polymorphism, eQTL, expression quantitative trait loci, se, standard error, BC, breast cancer, HEIDI, heterogeneity in dependent instruments, nsnp, number of SNPs for HEIDI test.

**Supplementary File 1g** Comparison of gene expression of SLC12A2 between breast cancer patients and controls in Expression Atlas

| **Experiment accession** | **Comparison (normal as reference)** | **Sample part** | **Log_2 fold change** | **Adjusted p-value** |
| --- | --- | --- | --- | --- |
| E-GEOD-68086 | 'breast carcinoma' vs 'normal' | blood platelet | -3.3 | 7.70E-24 |
| E-GEOD-31138 | invasive ductal carcinoma' vs 'normal' | breast | 2.5 | 0.02863135 |
| E-GEOD-45581 | 'non-inflammatory breast cancer' vs 'normal' | breast | -1.5 | 0.029033235 |
| E-GEOD-54002 | 'breast cancer' vs 'normal' | mammary gland | -1.4 | 1.23364E-05 |
| E-GEOD-38959 | 'breast cancer; breast' vs 'normal; breast' | breast | -1.3 | 0.017083027 |

**Supplementary File 1h** Traits associated with top SNP of SLC12A2 in PhenoScanner

| **SNP** | **hg19_coordinates** | **Effect_allele** | **Other_allele** | **Trait** | **efo** | **Study** | **pmid** | **year** | **beta** | **se** | **p** | **Direction** | **Dataset** |
| --- | --- | --- | --- | --- | --- | --- | --- | --- | --- | --- | --- | --- | --- |
| rs17764730 | chr5:127357526 | C | T | Lymphocyte count | EFO_0004586 | Astle W | 27863252 | 2016 | 0.02 | 0.0042 | 4.53E-09 | + | Astle-W_Blood-Cell-Traits_EUR_2016 |
| rs17764730 | chr5:127357526 | C | T | Red cell distribution width | EFO_0004586 | Astle W | 27863252 | 2016 | 0.14 | 0.0041 | 1.40E-265 | + | Astle-W_Blood-Cell-Traits_EUR_2016 |
| rs17764730 | chr5:127357526 | C | T | Basal metabolic rate | EFO_0007777 | Neale B | UKBB | 2017 | -0.02 | 0.0019 | 1.04E-15 | - | Neale-B_UKBB_EUR_2017 |
| rs17764730 | chr5:127357526 | C | T | Body fat percentage | EFO_0007800 | Neale B | UKBB | 2017 | 0.01 | 0.0022 | 5.46E-09 | + | Neale-B_UKBB_EUR_2017 |
| rs17764730 | chr5:127357526 | C | T | Cellulitis | EFO_0003035 | Neale B | UKBB | 2017 | 0.00 | 0.0003 | 1.72E-11 | - | Neale-B_UKBB_EUR_2017 |
| rs17764730 | chr5:127357526 | C | T | Forced vital capacity | EFO_0004312 | Neale B | UKBB | 2017 | -0.01 | 0.0023 | 3.55E-09 | - | Neale-B_UKBB_EUR_2017 |
| rs17764730 | chr5:127357526 | C | T | Impedance of leg left | - | Neale B | UKBB | 2017 | 0.06 | 0.0026 | 1.03E-115 | + | Neale-B_UKBB_EUR_2017 |
| rs17764730 | chr5:127357526 | C | T | Impedance of leg right | - | Neale B | UKBB | 2017 | 0.06 | 0.0026 | 6.90E-104 | + | Neale-B_UKBB_EUR_2017 |
| rs17764730 | chr5:127357526 | C | T | Impedance of whole body | - | Neale B | UKBB | 2017 | 0.03 | 0.0022 | 1.11E-34 | + | Neale-B_UKBB_EUR_2017 |
| rs17764730 | chr5:127357526 | C | T | Leg fat percentage left | - | Neale B | UKBB | 2017 | 0.02 | 0.0018 | 8.99E-33 | + | Neale-B_UKBB_EUR_2017 |
| rs17764730 | chr5:127357526 | C | T | Leg fat percentage right | - | Neale B | UKBB | 2017 | 0.02 | 0.0018 | 4.69E-32 | + | Neale-B_UKBB_EUR_2017 |
| rs17764730 | chr5:127357526 | C | T | Leg fat-free mass left | - | Neale B | UKBB | 2017 | -0.02 | 0.0019 | 9.37E-40 | - | Neale-B_UKBB_EUR_2017 |
| rs17764730 | chr5:127357526 | C | T | Leg fat-free mass right | - | Neale B | UKBB | 2017 | -0.03 | 0.0019 | 2.26E-44 | - | Neale-B_UKBB_EUR_2017 |
| rs17764730 | chr5:127357526 | C | T | Leg predicted mass left | - | Neale B | UKBB | 2017 | -0.02 | 0.0018 | 2.48E-39 | - | Neale-B_UKBB_EUR_2017 |
| rs17764730 | chr5:127357526 | C | T | Leg predicted mass right | - | Neale B | UKBB | 2017 | -0.03 | 0.0018 | 8.81E-44 | - | Neale-B_UKBB_EUR_2017 |
| rs17764730 | chr5:127357526 | C | T | Trunk fat-free mass | - | Neale B | UKBB | 2017 | -0.01 | 0.0018 | 8.69E-13 | - | Neale-B_UKBB_EUR_2017 |
| rs17764730 | chr5:127357526 | C | T | Trunk predicted mass | - | Neale B | UKBB | 2017 | -0.01 | 0.0018 | 1.68E-12 | - | Neale-B_UKBB_EUR_2017 |
| rs17764730 | chr5:127357526 | C | T | Varicose veins of lower extremities | - | Neale B | UKBB | 2017 | 0.00 | 0.0004 | 3.71E-13 | - | Neale-B_UKBB_EUR_2017 |
| rs17764730 | chr5:127357526 | C | T | Whole body fat-free mass | - | Neale B | UKBB | 2017 | -0.02 | 0.0018 | 8.77E-22 | - | Neale-B_UKBB_EUR_2017 |
| rs17764730 | chr5:127357526 | C | T | Whole body water mass | - | Neale B | UKBB | 2017 | -0.02 | 0.0018 | 2.33E-21 | - | Neale-B_UKBB_EUR_2017 |

**Supplementary File 1i** Causal association between systolic blood pressure (exposure) and risk of breast cancer (outcome)

| **Method** | **Number of SNPs** | **beta** | **se** | **OR** | **95% CI** | **p** |
| --- | --- | --- | --- | --- | --- | --- |
| GSMR | 521 | -0.0310 | 0.0171 | 0.969 | 0.938-1.002 | 0.0693 |
| MR Egger | 745 | -0.0095 | 0.0043 | 0.991 | 0.982-0.999 | 0.0295 |
| Weighted median | 745 | 0.0004 | 0.0017 | 1.000 | 0.997-1.004 | 0.8306 |
| Inverse variance weighted | 745 | -0.0002 | 0.0016 | 1.000 | 0.997-1.003 | 0.9178 |
| Simple mode | 745 | 0.0005 | 0.0061 | 1.000 | 0.989-1.013 | 0.9359 |
| Weighted mode | 745 | 0.0005 | 0.0038 | 1.000 | 0.993-1.008 | 0.8971 |

Abbreviations: se, standard error, SNP, single nucleotide polymorphism, OR odds ratio, CI confidence intervals
